# Twist1 Controls a Cell-Specification Switch Governing Cell Fate Decisions within the Cardiac Neural Crest

**DOI:** 10.1371/journal.pgen.1003405

**Published:** 2013-03-21

**Authors:** Joshua W. Vincentz, Beth A. Firulli, Andrea Lin, Douglas B. Spicer, Marthe J. Howard, Anthony B. Firulli

**Affiliations:** 1 Riley Heart Research Center, Herman B. Wells Center for Pediatric Research, Division of Pediatrics Cardiology, Departments of Anatomy, Biochemistry, and Medical and Molecular Genetics, Indiana University Medical School, Indianapolis, Indiana, United States of America; 2 Department of Biomedical Sciences, College of Osteopathic Medicine, University of New England, Biddeford, Maine, United States of America; 3 Department of Neurosciences and Program in Neurosciences and Neurological Disorders, University of Toledo Health Sciences Campus, Toledo, Ohio, United States of America; California Institute of Technology, United States of America

## Abstract

Neural crest cells are multipotent progenitor cells that can generate both ectodermal cell types, such as neurons, and mesodermal cell types, such as smooth muscle. The mechanisms controlling this cell fate choice are not known. The basic Helix-loop-Helix (bHLH) transcription factor Twist1 is expressed throughout the migratory and post-migratory cardiac neural crest. *Twist1* ablation or mutation of the Twist-box causes differentiation of ectopic neuronal cells, which molecularly resemble sympathetic ganglia, in the cardiac outflow tract. Twist1 interacts with the pro-neural factor Sox10 via its Twist-box domain and binds to the *Phox2b* promoter to repress transcriptional activity. Mesodermal cardiac neural crest *trans*-differentiation into ectodermal sympathetic ganglia-like neurons is dependent upon Phox2b function. Ectopic Twist1 expression in neural crest precursors disrupts sympathetic neurogenesis. These data demonstrate that Twist1 functions in post-migratory neural crest cells to repress pro-neural factors and thereby regulate cell fate determination between ectodermal and mesodermal lineages.

## Author Summary

During vertebrate development, a unique population of cells, termed neural crest cells, migrates throughout the developing embryo, generating various cell types, for example, the smooth muscle that divides the aorta and pulmonary artery where they connect to the heart, and the autonomic neurons, which coordinate organ function. The distinctions between neural crest cells that will form smooth muscle and those that will become neurons are thought to occur prior to migration. Here, we show that, in mice with mutations of the transcription factor *Twist1*, a subpopulation of presumptive smooth muscle cells, following migration to the heart, instead mis-specify to resemble autonomic neurons. Twist1 represses transcription of the pro-neural factor *Phox2b* both through antagonism of its upstream effector, Sox10, and through direct binding to its promoter. Phox2b is absolutely required for autonomic neuron development, and indeed, the aberrant neurons in *Twist1* mutants disappear when *Phox2b* is also mutated. Ectopic Twist1 expression within all neural crest cells disrupts the specification of normal autonomic neurons. Collectively, these data reveal that neural crest cells can alter their cell fate from mesoderm to ectoderm after they have migrated and that Twist1 functions to maintain neural crest cell potency during embryonic development.

## Introduction

Neural Crest Cells (NCCs) are multi-potent progenitor cells, which after delaminating from the dorsal lip of the neural tube and migrating throughout the developing embryo, can differentiate along either ectodermal or mesodermal lineages [1]–[4]. For example, a subset of NCCs, the cardiac NCCs (cNCCs) invades the aorticopulmonary cushions (APC) and septum (AoPS) of the developing cardiac outflow tract (OFT), the conduit through which blood exits the ventricles [5]–[8]. There, these cells assume a mesodermal identity, differentiating into connective tissue and smooth muscle and septating the pulmonary trunk and aorta to divide systemic and pulmonary circulation. Alternatively, the trunk, vagal, and sacral NCCs assume ectodermal identities, differentiating into the sympathetic, parasympathetic, and enteric neurons of the autonomic nervous system [9]. The transcriptional mechanisms that regulate NCC fate choice between these ectodermal and mesodermal lineages are not known. NCCs are specified along a rostro-caudal axis into distinct subpopulations that have limited capacity to change their cell fate [10]. However, both the cardiac and the rostral-most vagal neural crest originate at the same axial level (somites 1–3), suggesting that additional mechanisms of cell fate determination beyond axial specification are necessary to distinguish these ectodermal and mesodermal lineages. Indeed, both differentiating sympathetic neurons and cNCCs have been shown to respond to local secreted signaling cues, notably Bone Morphogenetic Proteins (BMPs), subsequently upregulating transcriptional effectors such as the Twist family bHLH proteins, Hand2 and Hand1, and initiating differentiation programs [11], [12]. The mechanisms that enable post-migratory NCCs to interpret these local signaling cues and to undergo either ectodermal or mesodermal differentiation programs are not understood.

In *Drosophila*, the transcription factor *twist* is essential for mesoderm development. In vertebrates; however, despite the evident requirement for Twist1 in the development of multiple mesodermally-derived organ systems, an analogous molecular mechanism by which Twist1 might control mesenchymal cell fate choice has not been defined [13]. Previously, we have shown that *Twist1^−/−^* knockouts display cNCC phenotypes at E11.5, including abnormally compacted cell aggregations in the APC ectomesenchyme. Cell-lineage and marker analyses show that these aggregations are NCC-derived and robustly express *Hand1* and *Hand2* [14]. We hypothesized that these mutant phenotypes reflect aberrant NCC fate choice, and sought to better define these NCC aggregates.

To precisely examine Twist1 function during cNCC maturation, we conditionally inactivated *Twist1* in NCCs at specific stages of *Mus musculus* (mouse) development using the *Wnt1-Cre* [15] and *Hand1^Cre^* [16] alleles, which respectively express *Cre recombinase* in pre-migratory and post-migratory cNCCs. Both cNCC-specific *Twist1* ablation models display OFT defects; however, pre-migratory *Twist1* deletion causes these defects with greater penetrance and severity, compared to post-migratory deletion. In contrast, the presence of NCC-derived nodules is evident in all *Twist1* conditional knockouts (CKOs), regardless of the timing of gene ablation. Expression analyses show that *Twist1* CKO nodules are molecularly similar to sympathetic ganglion neurons. We find that, unlike that of *bona fide* sympathetic ganglion neurons, ectopic neuron formation in *Twist1* CKOs is independent of *Hand2* function, indicating that these neurons are a distinct cell population. Similar to Hand1 and Hand2, Twist1 can molecularly interact with both the pro-neural Paired-like homeobox transcription factor Phox2b and HMG box transcription factor Sox10. Sox10 is upstream of *Phox2b* in the Bmp-dependent sympathetic neuron transcriptional program [17]. Here, we confirm that Sox10 regulation of *Phox2b* is direct. We demonstrate that Sox10 *trans*-activates the *Phox2b* promoter, and that Twist1 represses this transactivation. Twist1 itself can bind to an evolutionarily conserved non-canonical E-Box in the *Phox2b* promoter. Mutation of a C-terminal domain known as the Twist-box disrupts the ability of Twist1 to molecularly interact with Sox10, bind DNA, and transcriptionally repress *Phox2b*. Embryos harboring this Twist-box mutation (the *Charlie Chaplin Twist1* allele) [18] similarly display ectopic neurons in their OFTs. Indeed, the appearance of these ectopic neurons is dependent upon Phox2b, as *Phox2b* ablation rescues ectopic neuron formation in *Twist1* mutants. Finally, we show that ectopic *Twist1* expression in NCCs, using a conditionally activatable *CAG-CAT-Twist1* transgene activated by *Wnt1-Cre*, leads to sympathetic ganglia containing fewer neurons, in which Tyrosine Hydroxylase (TH), *Phox2b*, and *Hand2* expression is diminished or absent, demonstrating that Twist1 expression is sufficient to disrupt normal neurogenic developmental programs. Together, these data suggest that Twist1 represses neuronal cell fate choice in the cNCC by repressing transcription of *Phox2b*, and reveal a fundamental mechanism controlling ectodermal versus mesodermal cell fate choice in NCCs.

## Results

### A subpopulation of NCCs in the *Twist1 CKO* cardiac OFT expresses neuronal markers

**Figure 1:**
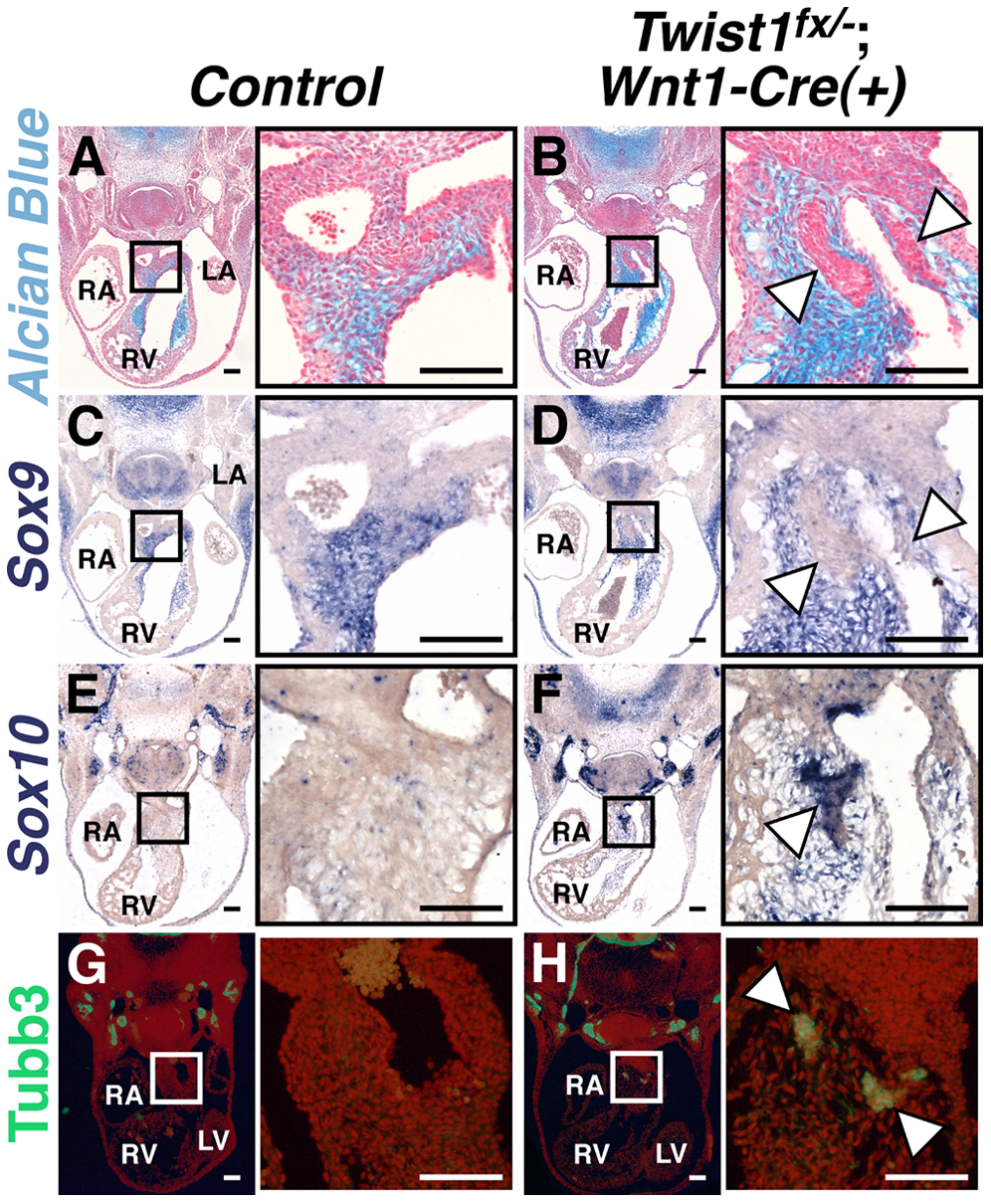
Abnormal NCCs in *Twist1*;*Wnt1-Cre* CKOs express neuronal, not mesenchymal, markers. A, B) Alcian blue staining to visualize ECM reveals that the cNCC-derived nodules (B, arrowheads) in E11.5 *Twist1^fx/−^*;*Wnt1-Cre(+)* mutants are devoid of ECM. C, D) *Sox9*, a transcriptional regulator of ECM components, is also absent from the nodules (D, arrowheads). E–H) Cell aggregates express the neuronal precursor marker *Sox10* (F, arrowheads) and neuronal marker Tubb3 (H, arrowheads). For all figures: LA, left atrium; LV, left ventricle; RA, right atrium; RV, right ventricle. Scale bars = 100 µm.

To assess Twist1 function during cNCC maturation, we conditionally inactivated *Twist1* in pre-migratory NCCs using the *Wnt1-Cre* driver. *Twist1 in situ* hybridization of E10.5 embryos confirmed effective *Wnt1-Cre*-mediated NCC-specific gene deletion (Figure S1). Conditional ablation of *Twist1* in pre-migratory NCCs produces abnormal cellular aggregates, indistinguishable from those observed in systemic *Twist1* knockouts [14], in the APCs of the cardiac OFT. Lineage trace analyses using the *ROSA26R* reporter allele confirms that these nodules are NCC-derived (data not shown) and establishes a cell-autonomous requirement for *Twist1* in developing cNCCs. At mid-gestation (E11.5), normal NCCs in the APCs generate extracellular matrix (ECM) that can be visualized by Alcian Blue staining (Figure 1A). The cNCC aggregations observed in *Twist1*;*Wnt1-Cre* CKO embryos are devoid of ECM (arrowheads, Figure 1B). *In situ* hybridization analysis demonstrates that expression of *Sox9*, a transcriptional regulator of ECM components, is abnormally absent from the aggregations (arrowheads, Figure 1D), but is expressed normally within non-phenotypic cNCCs. In addition to *Sox9*, *PlexinA2*, *Smad6*, *Hey2* and *Pdgfr*α, which are normally expressed in APC mesenchyme, are also excluded from the NCC aggregates (Figure S2). Thus, the cNCC aggregates in the cardiac OFT are molecularly distinct from the phenotypically normal cNCCs fated to differentiate along a mesodermal lineage and to ultimately contribute to the AoPS and valves. As reported previously, the feature that distinguishes these cNCC aggregates from normal cNCC mesenchyme is the expression of *Hand1* and *Hand2*, which accounts for approximately 30–40% of the cNCC within the E11.5 OFT [14]. Indeed, neither *Hand1* nor *Hand2* expression is readily detectible within non-aggregated cNCCs in *Twist1^−/−^* embryos [14].

As the NCC aggregations do not express the expected ecto-mesenchymal markers, we assessed the expression of genes associated with other NCC cell fates. The Bmp-dependent gene *Sox10* is expressed in NCC-derived neuronal progenitors [12]. *In situ* hybridization analysis demonstrates that *Sox10* mRNA is not detected in the APCs of control embryos; however, it is expressed in the *Twist1*;*Wnt1-Cre* CKO aggregates (Figure 1F). Immunohistochemical analyses revealed that these aggregates also express the pan-neuronal marker Tubulin β-III (Tubb3), confirming their identity as ectopic neurons (Figure 1H).

### Ectopic neurons in the *Twist1 CKO* OFT express sympathetic nervous system markers

**Figure 2:**
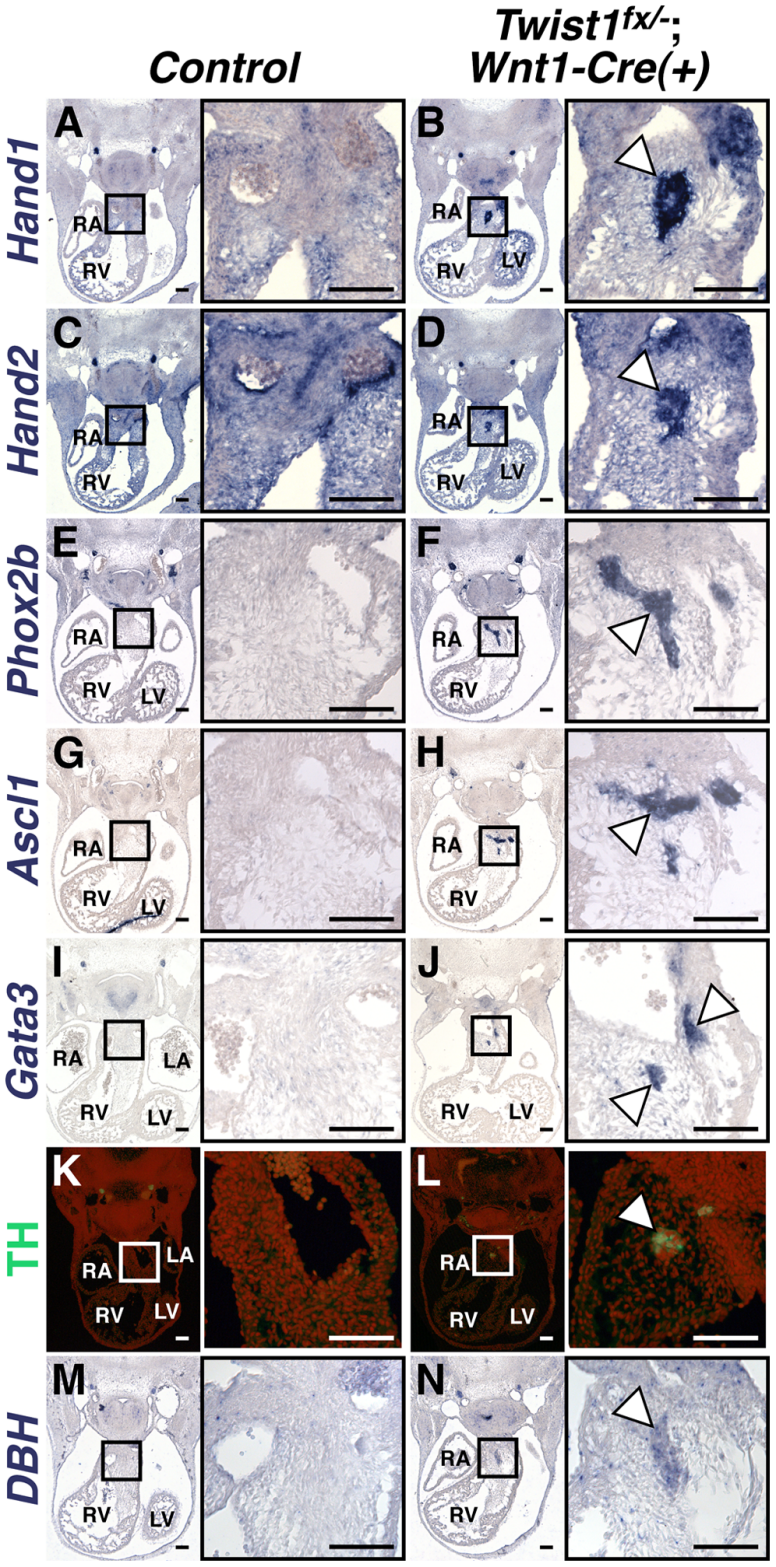
Ectopic neurons in *Twist1*;*Wnt1-Cre* CKO APCs express sympathetic neuron markers. *In situ* hybridization shows that, in *Control* E11.5 embryos, *Hand1* (A) and *Hand2* (C) expression is detectable throughout the cNCC-derived APCs, where as expression of other components of the sympathetic neurogenesis pathway, including *Phox2b* (E), *Ascl1* (G), *Gata3* (I), TH (K, as assessed via immunohistochemistry), and *DBH* (M) are not detectable in this tissue. E11.5 *Twist1^fx/−^*;*Wnt1-Cre(+)* mutants, aggregates (arrowheads) express the transcription factors *Hand1* (B), *Hand2* (D), *Phox2b* (F), *Ascl1* (H), and *Gata3* (J), and the norepinephrine biosynthetic enzymes TH (L), and *DBH* (N). (n = 3).

As NCCs contribute to a number of distinct neuronal populations, we sought to determine the cellular identity of the ectopic neurons in the *Twist1*;*Wnt1-Cre* CKO OFTs. Our previous data showed that *Hand1* and *Hand2*, both markers of cardiac NCCs, are readily detectable in the OFT aggregates of *Twist1^−/−^* mutant embryos [14]. We confirmed this finding in *Twist1*;*Wnt1-Cre* CKOs (Figure 2B, 2D). Importantly, *Hand1* and *Hand2* are only robustly co-expressed in NCC-derived sympathetic ganglion neurons (SGNs), and no other neuronal cell type [9], [19], [20]. We therefore hypothesized that these ectopic neurons may express other SGN markers. *In situ* hybridization revealed that NCC aggregates express transcriptional regulators of sympathetic neurogenesis, including the transcription factors *Phox2a* (data not shown) and *Phox2b* [21] (Figure 2F), the bHLH transcription factor *Ascl1* [22] (Figure 2H) and the zinc finger transcription factor *Gata3* [23] (Figure 2J). These cells also express the norepinephrine biosynthetic enzymes Tyrosine Hydroxylase (TH; Figure 2L) and *Dopamine β Hydroxylase* (*DBH*; Figure 2N) indicating that, like SGNs, these neurons are noradrenergic. Ectopic *Phox2b*- and *Ascl1*-positive NCCs are detectable in the *Twist1*;*Wnt1-Cre* CKO pharyngeal arches as early as E10.5, and persist at least until E16.5 (Figure S3).

To further confirm that these neurons most resemble sympathetic neurons, we assessed a panel of sensory neuron markers, including *TrkA*, *Brn3a*, *NeuroD1*, and *Runx1*. We found that ectopic OFT neurons do not express these sensory neuron markers (Figure S4).

We also sought to distinguish these ectopic neurons, which are noradrenergic, from parasympathetic neurons, which are predominantly cholinergic. We therefore analyzed the cholinergic neuron markers *Ret*, *VaCHT*, and *ChAT*. *Ret* and *VaCHT* are expressed in both parasympathetic neurons, and in sympathetic neurons during their early development [24], and both are detectable in ectopic OFT neurons (Figure S4). Conversely, the parasympathetic-specific marker *ChAT* is not detectable above background levels in ectopic OFT neurons (Figure S4). Given that *Hand1* and *Hand2* are not expressed within parasympathetic neurons [25], [26], we conclude that the ectopic neurons in the *Twist1*;*Wnt1-Cre* CKO APCs are most molecularly similar to sympathetic neurons.

In addition to the formation of ectopic OFT sympathetic-like neurons, *Twist1*;*Wnt1-Cre* CKOs also display completely penetrant persistent truncus arteriosus (PTA; Figure S5G, S5K; Table S1). These structural OFT defects are presaged by the complete absence of detectable *Semaphorin 3c* (*Sema3c*) expression in *Twist1*;*Wnt1-Cre* CKO cNCCs (Figure S6). *Sema3c* is required for septation of the OFT [27], [28] and may be directly regulated by Twist1 [29]. As PTA can result from either defective cell migration or NCC differentiation, a breakdown of either of these two mechanisms could cause the ectopic sympathetic-like neurons in the *Twist1*;*Wnt1-Cre* CKO APCs. To distinguish between these two possibilities, we deleted *Twist1* in post-migratory cNCCs.

### Twist1 functions in post-migratory cNCCs to repress neurogenesis

NCC fate choice is largely determined by axial level of origin [2], [4], [10]. *Twist1* is expressed in NCCs immediately following delamination from the neural tube and throughout subsequent migration. Loss of *Twist1* disrupts cranial NCC migratory pathways [30]. To test whether loss of *Twist1* either causes presumptive neural progenitor NCCs to similarly mis-migrate into the APC mesenchyme or alters cNCC fate choice, we examined *Twist1* function during post-migratory cNCC maturation. To this end, we conditionally inactivated *Twist1* using the *Hand1^Cre^* knock-in allele [16], which expresses *Cre* recombinase in post-migratory NCCs (Figure S5C, S6D). *Twist1*;*Hand1^Cre^* CKOs show a markedly reduced penetrance of PTA (Figure S5L, S5M; Table S1). This suggests that, for the OFT to septate properly, *Twist1* function is required to regulate cNCC migration to the OFT during early development.

**Figure 3:**
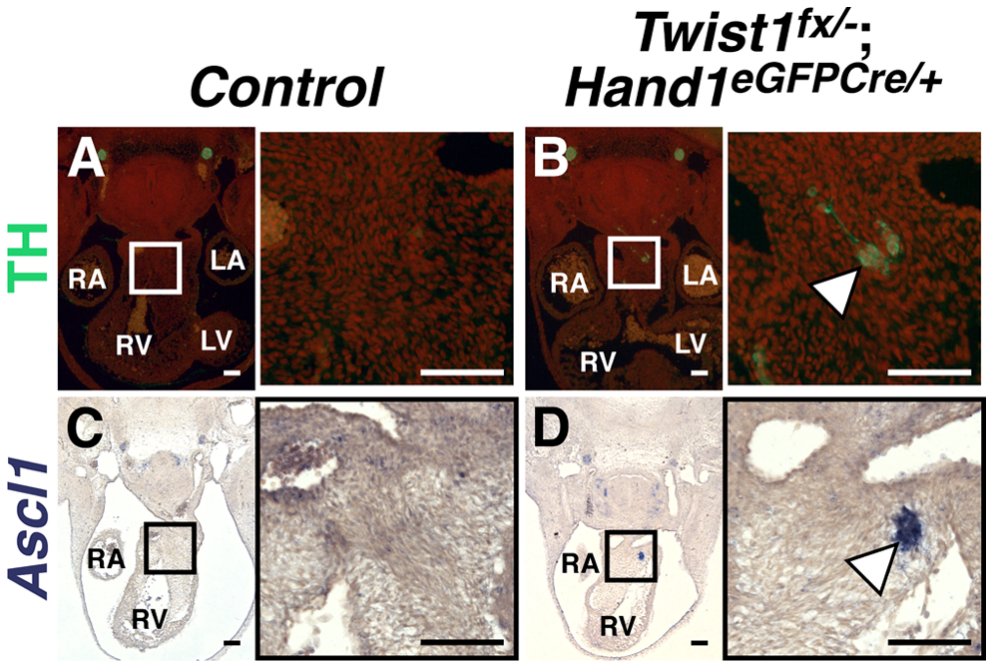
Ectopic sympathetic-like neurons are detectable in *Twist1*;*Hand1^eGFPCre^* CKO APCs. E11.5 *Twist1^fx/−^*;*Hand1^Cre^* mutant aggregates (arrowheads) express TH (B; n = 2) and *Ascl1* (D; n = 4), whereas these factors are not detectable in *Control* APCs (A and C, respectively).

In contrast, the formation of ectopic neurons in E11.5 *Twist1*;*Hand1^Cre^* CKO OFTs occurs with 100% penetrance (Figure 3B and 3D, arrowheads). *In situ* hybridization and immunohistochemical analyses show that ectopic neurons express *Sox10*, Tubb3 (data not shown), TH (Figure 3B) and *Ascl1* (Figure 3D). Thus, when Twist1 function is ablated in *Hand1*-lineage cNCCs that have completed migration, a subset of these cells then differentiate into neurons. These data indicate that ectopic neurons form in *Twist1* mutant embryos via a novel post-axial specification mechanism whereby post-migratory cNCCs *trans*-differentiate. Additionally, although *Sema3c* expression was also disrupted in a majority of *Twist1*;*Hand1^Cre^* CKO OFTs (n = 3/4), unlike *Twist1*;*Wnt1-Cre* CKOs, the penetrance was not 100%. Indeed, *Sema3c* expression was unaffected, even when ectopic *Ascl1*-positive cells were detectable (Figure S6). Collectively, these data demonstrate that OFT septation and repression of cNCC *trans*-differentiation are distinct Twist1-associated phenotypes, and that this *Hand1*-lineage-resticted *trans*-differentiation does not preclude the remaining cNCCs from maintaining characteristic cNCC gene expression (Figure S2) and differentiating normally once they arrive within the OFT.

### A non-canonical pathway specifies ectopic sympathetic-like neurons in the *Twist1 CKO* OFT

**Figure 4:**
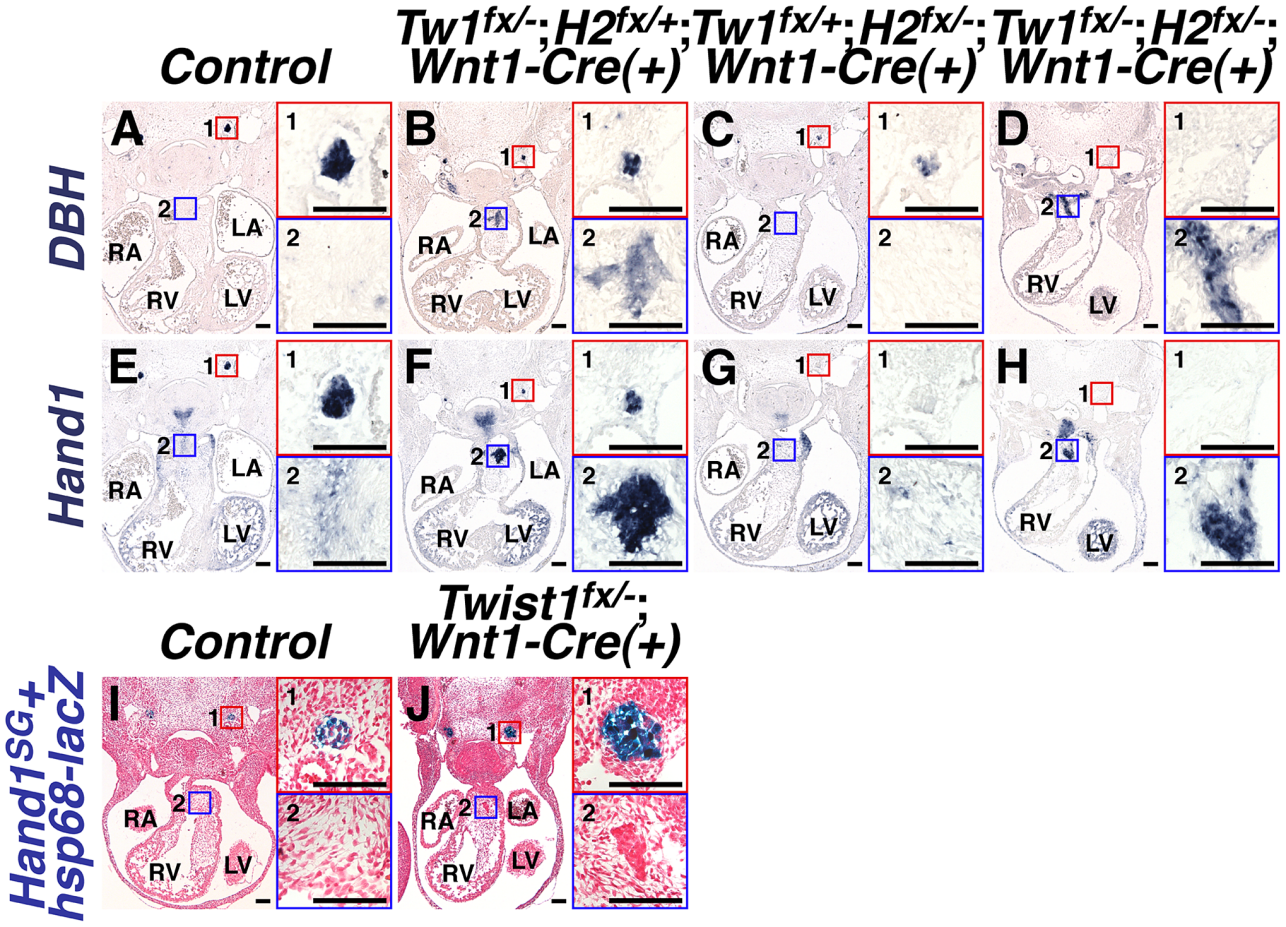
Ectopic sympathetic-like neurons in *Twist1*;*Wnt1-Cre* CKO APCs are not *bona fide* sympathetic neurons. A–H) At E11.5, NCC-specific *Hand2* ablation causes reduced *DBH* expression (compare inset 1 in A and C), and loss of *Hand1* expression (compare inset 1 in E and G). However, in E11.5 *Twist1^fx/−^*;*Hand2^fx/−^*;*Wnt1-Cre(+)* mutants, robust ectopic sympathetic-like neurons persist and express comparatively high levels of *DBH* (inset D2; n = 4) and *Hand1* (inset H2; n = 4). I, J) A Hand2-dependent *cis*-regulatory element proximal to *Hand1* is sufficient to drive reporter transgene (*Hand1^SG^+hsp68-lacZ*) expression in developing SGNs at E11.5 (inset 1 in I and J). However, reporter transgene expression is not detectable in the ectopic neurons in the *Twist1^fx/−^*;*Wnt1-Cre(+)* CKO APCs (J2), indicating that this tissue-specific *cis*-regulatory element is not active in these cells (n = 4).

To identify the transcriptional effectors of sympathetic neurogenesis that Twist1 regulates, we first explored genetic interactions with the related bHLH factor *Hand2*. Sympathetic neurogenesis depends upon Hand2 function [26], [31], [32]. As Twist1 and Hand2 are functionally antagonistic in the developing limb [33] we investigated a similar mechanism in the OFT. As expected, at E11.5, NCC-specific *Hand2* ablation results in fewer *DBH*-positive neurons (compare Figure 4A1 and 4C1), reduced TH expression (data not shown), and complete loss of *Hand1* expression (compare Figure 4E1 with 4G1) in the forming SGNs. *Twist1*;*Hand2* double CKOs display similarly reduced SGN specification (Figure 4D1 and 4H1). Surprisingly, ectopic sympathetic-like neurons persist in the OFTs of E11.5 *Twist1*;*Hand2* double CKOs (Figure 4D and 4H). These ectopic neurons robustly express TH (data not shown), *DBH* (Figure 4D2), and *Hand1* (Figure 4H2). Endogenous *Hand1* SGN expression is directly dependent upon Hand2 function [26], [32], [34]. Although the ectopic neurons in the *Twist1* CKO APCs are molecularly similar to SGNs, their specification and expression of SG-specific markers is Hand2-independent, thereby distinguishing them as a separate neuronal cell population. Thus, Twist1 antagonism of Hand2 does not repress ectopic neurogenesis in developing cNCCs.

We have identified a Hand2-dependent *Hand1 cis*-regulatory enhancer sufficient to drive reporter gene expression in the developing and adult SGNs [34]. To validate that the ectopic neurogenesis in *Twist1* CKO APC mesenchyme is truly via a non-canonical mechanism, we assessed the expression of this SGN-specific enhancer (*Hand1^SG^+hsp68-lacZ*) on a *Twist1*;*Wnt1-Cre* CKO background. Although this SGN-specific reporter transgene is normally expressed by the endogenous E11.5 SGNs of both control and *Twist1*;*Wnt1-Cre* CKO embryos (Figure 4, 4I1 and 4J1), the *Hand1* SGN-specific enhancer is not transcriptionally active in *Twist1*;*Wnt1-Cre* CKO ectopic neurons, despite the presence of its direct regulators, Hand2 and Phox2b [34] Figure 4J2). Thus, although the ectopic neurons in *Twist1*;*Wnt1-Cre* CKO APCs express *Hand1* (Figure 2B; Figure 4F2; [14], this expression is driven by a Hand2-independent enhancer distinct from the one that drives SGN-specific expression. Thus, the molecular pathways regulating gene expression in the ectopic neurons of *Twist1*;*Wnt1-Cre* CKO APCs are distinct from those endogenously regulating sympathetic neurogenesis.

Interestingly, Hand2-independent *Hand1* expression in the *Twist1*;*Wnt1-Cre* CKO ectopic OFT neurons suggests that Hand1 could be functionally redundant with Hand2, and that loss-of-function of both *Hand1* and *Hand2* would “rescue” the ectopic neurons in *Twist1*;*Wnt1-Cre* CKOs. To test this, we generated *Twist1^fx/−^*;*Hand1^fx/−^*;*Hand2^fx/−^*;*Wnt1-Cre(+)* triple CKOs (Figure S7). Triple CKO embryos were rarely viable past E10.5. Nevertheless, histological analysis clearly shows ectopic OFT neurons, further confirming that, although the 30–40% of cNCC undergoing neuronal *trans*-differentiation are marked by *Hand1* and *Hand2* [14], this aberrant NCC cell fate *trans*-differentiation caused by *Twist1* loss-of-function is completely Hand factor independent.

### Twist1 molecularly interacts with both Phox2b and Sox10 through a protein domain required to repress ectopic neurogenesis in the OFT

As Hand factors are dispensable for ectopic neuron formation in the *Twist1* CKO APCs, we sought an alternative pro-neural factor(s) with which Twist1 interacts to repress neurogenesis. Hand2 and Phox2 proteins molecularly interact to synergistically affect critical transcriptional programs during sympathetic neurogenesis [26], [31], [32], [35]. Phox2b is molecularly upstream of Hand2, and these two factors auto-regulate each other via a feed-forward mechanism [21], [22], [36]. As Twist1 and Hand2 are closely related, we used co-immunoprecipitation to test for a possible interaction between Twist1 and Phox2b. Similar to Hand2, Twist1 molecularly interacts with Phox2b (Figure S8A and S8B). Twist1 molecularly interacts with both Runx2 [18] and Runx3 [36] through its carboxy-terminal Twist-box domain. Mutation of the Twist-box (S192P) impairs Runx molecular interactions. [18] Co-immunoprecipitation analyses using epitope-tagged Twist1 S192P showed that protein interactions between Twist1 and Phox2b are, in part, dependent upon the Twist-box domain (Figure S8A and S8B). However, transactivation assays in HeLa cells revealed that Twist1 does not significantly inhibit Phox2b auto-regulation, and thus did not provide compelling evidence that Twist1-mediated antagonism of neurogenesis is mediated though direct inhibition of Phox2b protein function. We therefore sought additional pro-neural factors with which Twist1 might interact.

**Figure 5:**
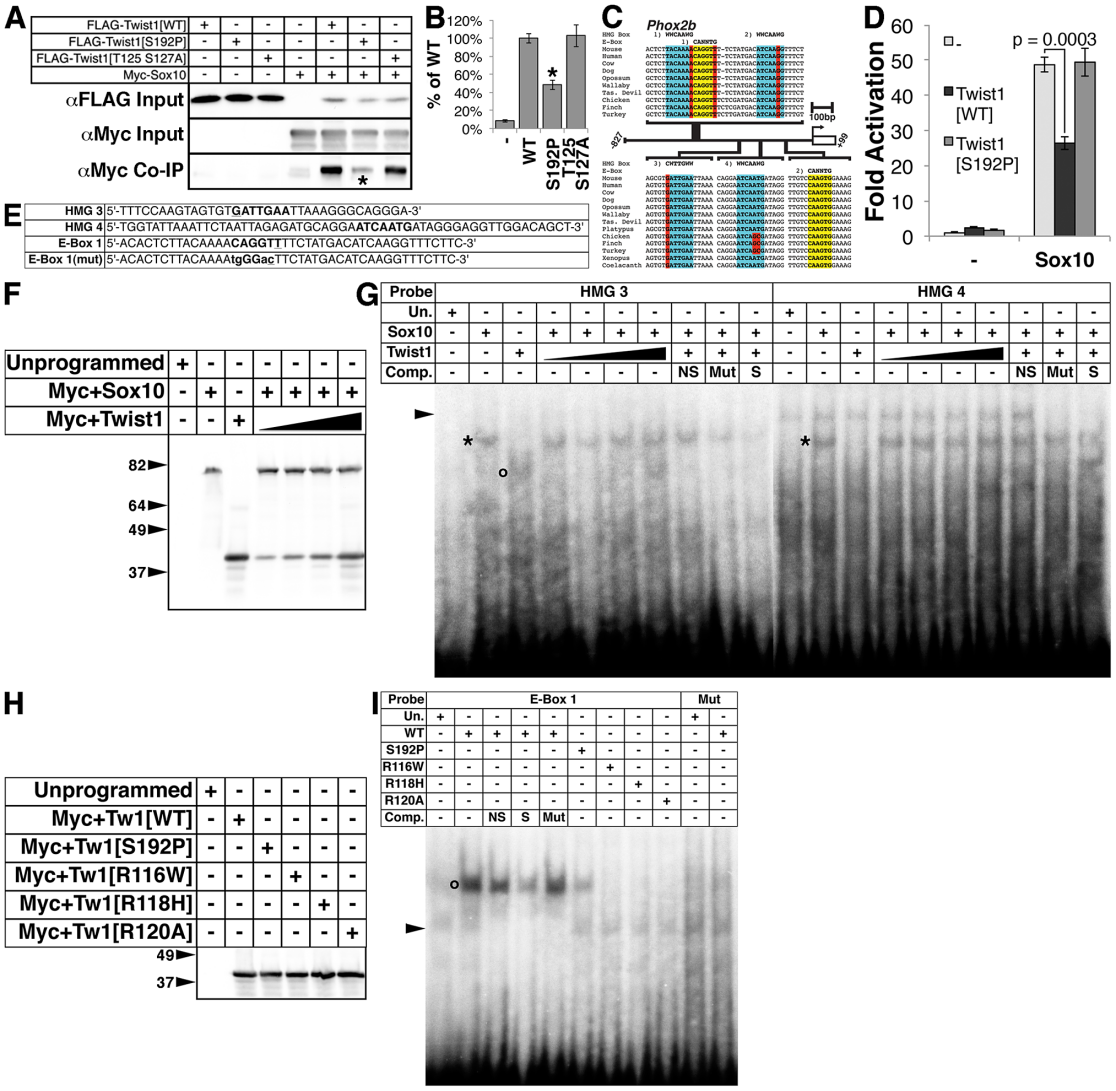
The Twist-box domain of Twist1 is required for molecular interaction with Sox10 and for Twist1 binding to the *Phox2b* promoter. A) Co-immunoprecipitation assays (n = 3) using epitope-tagged variants of Twist1 and Sox10 show that Twist1 molecularly interacts with Sox10. This interaction is diminished in the Twist1 S192P mutant (asterisk), but not the Twist1 T125;S127A mutant. B) Densitometry analyses quantitate these results. C) Bioinformatic analyses uncovered four evolutionarily conserved HMG Box binding sites (highlighted in blue) and two conserved E-Box binding sites (highlighted in yellow) within in the 827 bps 5′ to the *Phox2b* transcription start site. Nucleotides that diverge from established consensus sequences are highlighted in red. D) Transactivation assays of the *Phox2b* promoter show that Sox10 upregulates gene expression from the *Phox2b* promoter 48.9+/−2.2-fold. Transcriptional upregulation is significantly reduced to 26.3+/−1.9-fold (p-value = 0.0003) when Sox10 is co-transfected with Twist1. When Sox10 is co-transfected with the Twist1 S192P mutant, Twist1 repression of Sox10 activity on the *Phox2b* promoter is lost (49.3+/−3.9-fold; p-value = 0.92). E–I) Both (E) radiolabeled oligonucleotides containing HMG Box or E-Box consensus sites (bold) and (F) reticulocyte lysates programmed with Myc+Sox10 and/or Myc+Twist1 were used to confirm that (G) Sox10 directly binds to *cis*-elements within the *Phox2b* promoter (asterisks), and that Twist1 has no appreciable effect upon Sox10 DNA binding. An open circle denotes a complex formed between HMG 3 and Twist1. H) A Western blot for α-Myc verifies that the Myc-tagged Twist1 variants transcribed *in vitro* were synthesized in equivalent amounts. I) EMSAs employing these lysates revealed that E-Box 1 is bound robustly by wild-type Twist1 (open circle), weakly by Twist1 S192P, and not at all by Twist1 R116W, Twist1 R118H, or Twist1 R120A. Mutation of the E-Box, E-Box 1(Mut), completely ablated Twist1 binding. Underlined nucleotides in oligo sequences indicate those that diverge from established consensus sequences. Mutated nucleotides are in lower case. Arrowheads in EMSAs denote non-specific binding. NS, non-specific unlabeled competitor; Mut, mutant unlabeled competitor; S, specific unlabeled competitor.

*Phox2b* is not normally expressed in wild-type APCs (Figure 2E). Its presence in the APCs of *Twist1* mutant embryos suggests that Twist1 could either directly repress *Phox2b* expression or repress the activity of a key Phox2b transcriptional regulator. Recently, Twist1 was shown to interact with and repress the function of the transcription factor Sox9 [37]. The related factor Sox10 functions directly upstream of *Phox2b* in the Bmp-dependent pathway that drives sympathetic neurogenesis [17]. To assess whether Twist1 can also interact with Sox10 and potentially inhibit its transcriptional activity, we performed co-immunoprecipitation assays. Twist1 molecularly interacts with Sox10, and although Twist1 S192P displays a comparatively reduced interaction with Sox10 (Figure 5A, 5B, asterisk, 48.3%+/−5.1%, p-value = 0.002), Twist1 T125;S127A, a Saethre-Chotzen Syndrome-associated Helix I dimerization mutant, does not (Figure 5A, 5B, 102.7%+/−12.0%, p-value = 0.85). Thus, as has been reported for Sox9 [37], Sox10 also interacts with Twist1 in a Twist-box dependent manner.

Sox proteins bind the consensus sequence WWCAAWG [38]. Bioinformatic analyses revealed putative HMG box *cis*-elements in the *Phox2b* 5′ promoter that are evolutionarily conserved (Figure 5C, highlighted in blue). Sox10 is required for *Phox2b* expression *in vivo* [17]. Given the presence of evolutionarily conserved putative Sox10 *cis*-elements in the *Phox2b* promoter (Figure 5C), we tested whether Sox10 might transactivate *Phox2b* directly. Indeed, Sox10 significantly upregulates gene expression from the *Phox2b* promoter by 48.9+/−2.2-fold (Figure 5D, p-value = 0.0002). Co-expression of Twist1 inhibits Sox10-mediaded transactivation, reducing its transcriptional activation to 26.3+/−1.9-fold (Figure 5D, p-value = 0.0003). Co-expression of Twist1 S192P significantly ameliorates this Sox10 inhibition, restoring Sox10 transactivation to 49.3+/−3.9-fold (Figure 5D, p-value = 0.92). Together, these data indicate that Twist1 molecularly interacts with Sox10 through its Twist-box domain, and partially represses *trans*-activation of the *Phox2b* promoter.

Twist1, through its Twist-box domain, inhibits DNA binding of both Runx2 [18] and Sox9 [37]. We therefore reasoned that Twist1 might similarly inhibit Sox10 binding to the *Phox2b* promoter. We therefore first confirmed Sox10 DNA binding to the conserved HMG boxes in the *Phox2b* 5′ promoter through electrophoretic mobility assays (EMSA) using radiolabeled oligonucleotides (Figure 5E) and *in vitro* translated Myc-tagged Sox10 (Figure 5F). Sox10 binds to the proximal two HMG boxes, termed HMG 3 and HMG 4 (Figure 5G, asterisks), but not the distal two HMG boxes, termed HMG 1 and HMG 2 (data not shown). We conclude that Sox10 can bind to conserved HMG boxes in the *Phox2b* promoter, supporting that Sox10 directly regulates *Phox2b* expression. We then synthesized *in vitro* translated Myc-tagged Sox10 in the presence of increasing amounts of Myc-tagged Twist1 (Figure 5F). EMSAs revealed that Twist1 does not disrupt Sox10 DNA binding to HMG 3 and HMG 4 (Figure 5G, asterisks). However, Twist1 does bind within HMG 3 to a non-canonical *cis*-element (Figure 5G, open circle). Given this surprising result, we then tested whether Twist1 can bind directly to other elements within the *Phox2b* promoter.

Twist1 binds to consensus sites termed E-Boxes (CANNTG) [39], [40]. Bioinformatic analyses uncovered one conserved E-Box and one conserved E-Box-like element within the *Phox2b* 5′ promoter (Figure 5C, highlighted in yellow). We *in vitro* translated Twist1 and Twist1 S192P protein (Figure 5H) to assess Twist1 DNA binding to these E-Boxes. As controls, we included three mutant forms of Twist1 in which conserved arginines in the basic domain have been mutated (Figure 5H). Of these three mutants, Twist1 R116W [41], Twist1 R118H [42], and Twist1 R120A [43], the former two are associated with the congenital disorder Saethre-Chotzen Syndrome, and all are predicted to have impaired DNA binding capabilities [44]. Although Twist1 displayed no binding to the perfect consensus E-Box 2 (data not shown), it robustly bound to the non-canonical E-Box 1 (Figure 5I, open circle). Twist1 did not detectably bind to a mutated (mut) E-Box 1 oligo (Figure 5I), in which the E-Box core was disrupted (Figure 5I). As predicted, none of the Twist1 basic domain mutants bound to E-Box 1. Twist1 S192P does bind to E-Box 1; however, it binds more weakly when compared to WT Twist1. Given that equivalent amounts of Twist1 and Twist1 S192P were added to each EMSA, the strength of DNA-binding is quantitative (see [39]; Figure 5H and 5I). Together, these data suggest that the Twist-box is critical not only for Twist1 protein interactions with Sox10, but also for Twist1 DNA binding. Thus, Twist1 inhibition of *Phox2b* transcription is likely mediated through direct DNA binding of non-canonical, conserved E-Box elements in the *Phox2b* promoter. Moreover, Twist1 could directly interact with the potent *Phox2b trans*-activator Sox10 while both factors are bound to the promoter.

**Figure 6:**
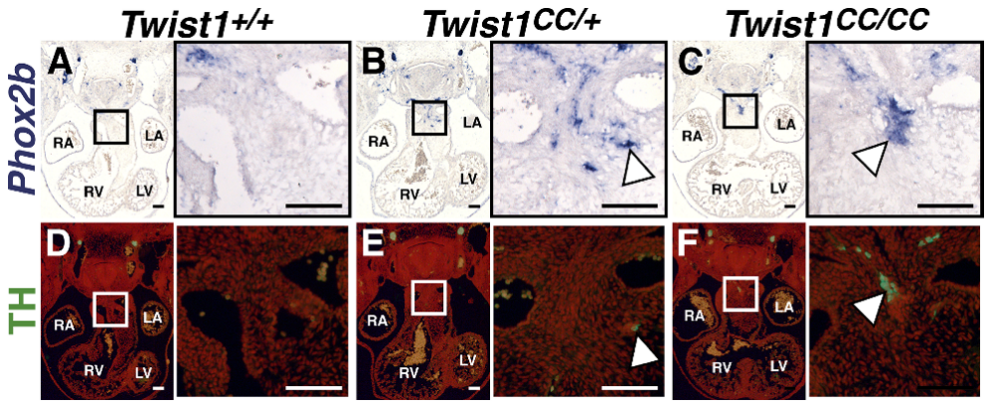
The Twist-box domain of Twist1 is required for repression of ectopic neurogenesis. Analyses of *Twist1^CC^* mutant embryos that harbor this Twist box mutation, reveal that *Twist1^CC/+^* heterozygous OFTs contain dispersed or relatively small aggregates of *Phox2b*-positive cells (B) and rare TH-positive cells (E, arrowhead) *Twist1^CC/CC^* embryos contain robust ectopic *Phox2b*-positive ganglia (C) and a greater number of TH-positive neurons (F, arrowhead). Neither *Phox2b*- nor TH-positive cells are detectable in *Twist1^+/+^* APCs (A and D, respectively).

**Figure 7:**
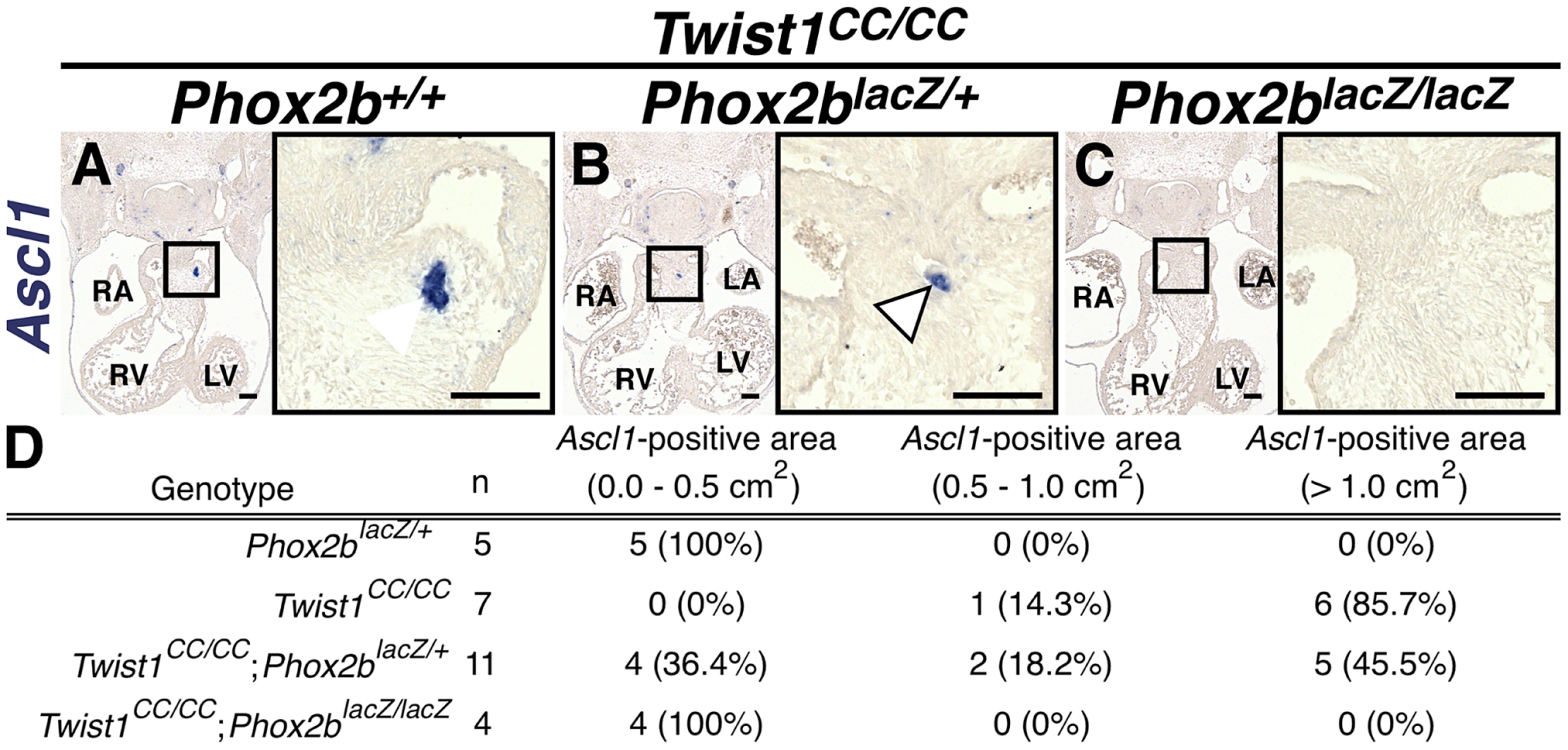
Ectopic neurogenesis in *Twist1^CC^* mutants is Phox2b-dependent. A) All *Twist1^CC/CC^* homozygous OFTs examined contain robust aggregates of *Ascl1*-positive cells. B) Concurrent *Phox2b^lacz/+^* haploinsufficiency tended to reduce the size of these aggregates (arrowhead). C) No *Ascl1*-positive cells were evident in *Twist1^CC/CC^*;*Phox2b^lacz/lacZ^* OFTs. D) Morphometric quantification of these analyses validated these results.

As Phox2b is considered a master regulator of autonomic neurogenesis, [9] we hypothesized that disruption of the Twist-box *in vivo* would lead to aberrant *Phox2b* activation, and, consequently, the appearance of ectopic neurons in the developing OFT similar to those observed in *Twist1* CKO embryos. The *Charlie Chaplin Twist1* allele (*Twist1^CC^*) is a *Twist1* S192P point mutation that specifically disrupts function of the *Twist1* Twist-box domain. Embryos harboring this mutant allele exhibit craniofacial and limb abnormalities [18]. To test the hypothesis that Twist1-mediated repression of *Phox2b* is necessary to repress ectopic neurogenesis in the developing OFT, we examined the OFT phenotypes of both heterozygous and homozygous *Twist1^CC^* embryos. *Twist1^CC/CC^* mutants lack overt structural OFT phenotypes (Figure S5I, S5M). Indeed, *Sema3c* expression is not disrupted in either *Twist1^CC/+^* or *Twist1^CC/CC^* mutants (Figure S6F, S6G). These data suggest that the Twist-box is dispensable for proper cNCC migration and OFT morphogenesis. However, as predicted by the necessity of the Twist-box for robust Sox10 interactions and binding to the *Phox2b* promoter, ectopic OFT neurons are detectable in the APCs of both *Twist1^CC/CC^* homozygous mutants and *Twist1^CC/+^* heterozygotes. In *Twist1^CC/+^* heterozygous embryos, dispersed *Phox2b*- (Figure 6B) and *Ascl1*-positive (data not shown) cells are detectable in the APC ectomesenchyme. Relatively few of these cells are TH-positive, however, suggesting that these ectopic cells fail to undergo complete neuronal differentiation (Figure 6E, arrowhead). *Twist1^CC/CC^* homozygous mutants display more robust ectopic ganglia that are positive for *Phox2b* (Figure 6C), *Ascl1* (Figure 7A), and TH (Figure 6F). Collectively, these data show that Twist1 interacts with Sox10 and Phox2b at least partially via its Twist-box domain, and that this domain is required to inhibit cNCC *trans*-differentiation into ectopic sympathetic-like neurons.

### Ectopic neurogenesis in *Twist1* mutants is Phox2b-dependent

To confirm that *Phox2b* upregulation is absolutely required to modulate post-migratory NCC cell fate choice in *Twist1* mutants, we assessed ectopic neurogenesis in E11.5 *Twist1*;*Phox2b* doubly mutant embryos. Robust ectopic aggregates of *Ascl1*-expressing cells were evident in *Twist1^CC/CC^* homozygous mutants (Figure 7A). Interestingly, the size of these aggregates appeared reduced when *Phox2b* gene dosage was attenuated to heterozygosity (Figure 7B). It is not technically possible to count individual staining-positive cells following *in situ* hybridization. We therefore validated our results using morphometric analyses. Quantification of the *Ascl1* staining-positive area in each sequential section of these mutant embryos revealed that, while a reduction of *Phox2b* gene dosage had no appreciable effect on *Ascl1* staining in 5 out of 11 *Twist1^CC/CC^*;*Phox2b^lacZ/+^* mutants examined, 4 out of 11 of these mutants showed reduced ectopic *Ascl1* staining comparable to that seen in control (*Phox2b^lacZ/+^*) APCs (Figure 7D). Ectopic neurons were not detectable in either *Twist1^CC/+^* or *Twist1^CC/CC^* mutants when *Phox2b* function was completely ablated (Figure 7C, data not shown). Collectively, these results validate the hypothesis that Twist1 ultimately represses the proneural activity of Phox2b in NCCs by inhibiting its transcription both via direct molecular antagonism of Sox10 activity and through binding to the *Phox2b* promoter. These findings provide the first evidence, to our knowledge, that the potential ectodermal and mesodermal cell fates of post-migratory NCCs can be regulated through functional antagonism between transcription factors.

### Ectopic expression of *Twist1* disrupts sympathetic neurogenesis

**Figure 8:**
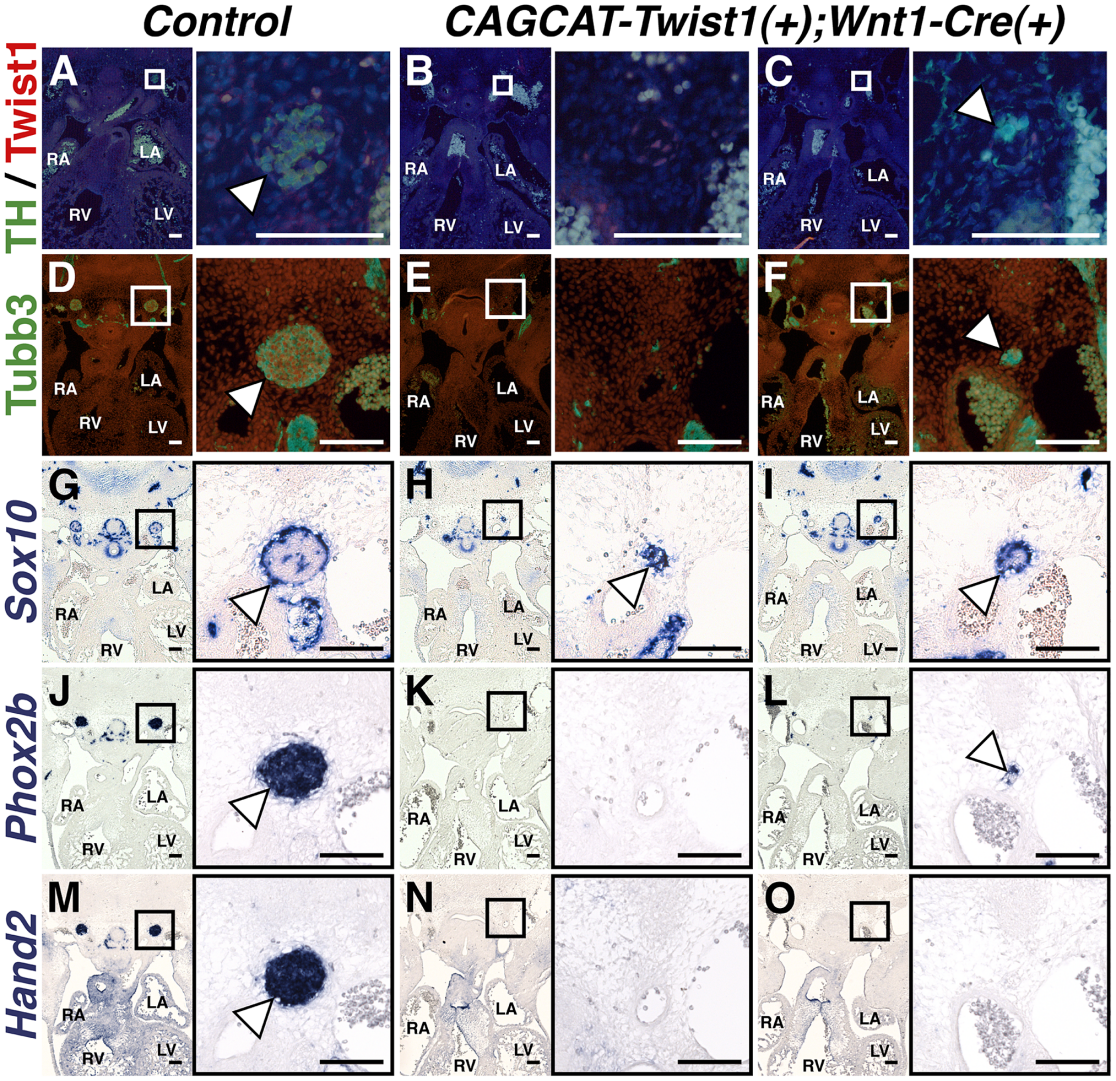
Sympathetic neurogenesis is disrupted by Twist1 mis-expression. In E12.5 Control embryos, the thoracic sympathetic chain ganglia (arrowheads) express TH (A), Tubb3 (D), *Phox2b* (J), and *Hand2* (M). *Sox10*-expressing support cells surround the thoracic sympathetic chain ganglia (G, arrowhead). In thoracic sympathetic chain ganglia in E12.5 *CAGCCAT-Twist1(+)*;*Wnt1-Cre(+)* transgenic embryos, expression of TH, Tubb3, *Phox2b*, and *Hand2* is either absent (B, E, K, and N), or markedly reduced (C, F, L, and O). In the latter instance, TH (green) and Twist1 (red) expression is largely mutually exclusive (n = 3). These hypoplastic thoracic sympathetic chain ganglia are either entirely positive for *Sox10* (H, arrowhead) or display a markedly reduced core of *Sox10*-negative cells (I, arrowhead).

If Twist1 is a true repressor of ectodermal cell fate, then ectopic expression of Twist1 in NCCs should inhibit differentiation of endogenous SGNs. To test whether Twist1 can repress sympathetic neurogenesis, we used a conditionally activatable transgene (*CAG-CAT-MycTwist1*) [40] to ectopically express *Twist1* in the NCC progenitors of SGNs. TH expression, as revealed through immunohistochemistry of E12.5 embryos, was either absent in thoracic sympathetic chain ganglia (Figure 8B), or was restricted to a few cells (Figure 8C). Co-localization of Twist1, visualized via a Myc epitope tag, and TH was not observed (Figure 8C). Tubb3 immunohistochemistry and *Phox2b in situ* hybridization analyses confirm that the thoracic sympathetic ganglia in *CAG-CAT-MycTwist1(+)*;*Wnt1-Cre(+)* embryos are either absent (Figure 8E, 8K), or markedly reduced (Figure 8F, 8L). In the *Control* thoracic sympathetic chain, *Sox10*-expressing presumptive support cells surround the ganglia, which are mostly, but not entirely, *Sox10*-negative (Figure 8G). In E12.5 *CAG-CAT-Twist1(+)*;*Wnt1-Cre(+)* transgenic embryos, the hypoplastic thoracic sympathetic chain ganglia are either entirely positive for *Sox10* (Figure 8K, arrowhead) or display a markedly reduced core of *Sox10*-negative cells (Figure 8I, arrowhead). *Hand2* mRNA expression is also drastically reduced in E12.5 *CAG-CAT-Twist1(+)*;*Wnt1-Cre(+)* thoracic sympathetic chain ganglia, and is occasionally absent, even when neurons in adjacent sections express *Phox2b*, suggesting that normal SGN regulatory cascades are disrupted in *Twist1* mis-expressing SGNs (Figure 8N, 8O). These findings, in combination with *Twist1* loss-of-function and genetic interaction analyses, demonstrate that Twist1 is a potent repressor of sympathetic neurogenesis, and that Twist1 antagonizes downstream Bmp targets to act as a novel post-migratory cell fate switch in the NCCs that populate the cardiac OFT.

## Discussion

Here, we describe a novel cell fate switch that regulates post-migratory NCC differentiation to either an ectodermal or a mesodermal cell fate. The bHLH transcription factor Twist1 is expressed in migratory and post-migratory cNCCs, but is not expressed in the NCC-derived SGNs. Loss of *Twist1* in *Wnt1-Cre*-expressing NCCs results not only in structural OFT defects, but also the formation of dense aggregates of sympathetic-like ganglia in the APCs of the cardiac OFT. These neurons express *Sox10*, *Phox2b*, *Ascl1*, *Gata3*, *Hand1*, *Hand2*, TH and *DBH*, which are all components of the BMP-induced SGN differentiation program, but not markers of sensory neurons or specific markers of parasympathetic neurons. Our earlier work shows that these ectopic neurons are marked by both *Hand1* and *Hand2* expression, and account for approximately 30–40% of E11.5 OFT cNCCs [14]. Deletion of *Twist1* in the smaller, post-migratory *Hand1^Cre^* lineage-derived subpopulation of cNCCs reveals that this ectopic neurogenesis is a *bona-fide* cell fate *trans*-differentiation from a mesenchymal to neuronal cell type, and is not a consequence of the altered migration of trunk NCCs. The penetrance of OFT structural defects, such as PTA, is greatly reduced in embryos in which *Twist1* is deleted using the *Hand1^Cre^*, indicating that NCC migration-dependent phenotypes are largely rescued in this model (Table S1). Thus, cells fated to contribute to the smooth muscle and valves of the OFT can be reprogrammed to an SGN-like fate in the absence of Twist1. Both *Wnt1-Cre* and *Hand1^Cre^ Twist1* CKOs survive until birth and, in some *Hand1^Cre^* Twist1 CKOs, exhibit no significant OFT defects other than ectopic neurons. Furthermore, although they have completely penetrant ectopic neurons, *Twist1^CC^* mutants do not display either structural OFT defects or diminished *Sema3c* expression. In conjunction with normal marker analysis (Figure S2), these data suggest that Twist1 function is solely required in specific subpopulations of cNCCs, and that unaffected cNCCs correctly follow their developmental programs in the absence of Twist1.

Mechanistically, Twist can physically interact with other proteins using both its bHLH and Twist-box domains [18], [37], [45], [46]. Twist1 antagonistically interacts with Hand2 [33]. Although loss of *Hand2* in NCCs dramatically effects the development of the endogenous sympathetic chain, as previously reported [26], [32], the *trans*-differentiation of the SGN-like neurons in the OFT was not affected. Furthermore, *Hand1* expression, which is directly dependent upon Hand2 function in SGNs [34], is maintained, but its expression is not mediated through its identified SGN-specific enhancer (Figure 4). Although we have demonstrated that the *Hand1* SG-enhancer is not auto-regulated by Hand1 [34], we analyzed *Twist1*;*Hand2*;*Hand1* triple CKO mice. Ectopic neurons remain histologically evident in these mutants, demonstrating that the *trans*-differentiation of cNCCs to an SGN-like fate is independent of Hand factors and, by consequence, represents a non-canonical transcriptional program. Hand factor expression, instead, appears to define which cNCCs convert to ectopic neurons, as opposed to those that retain their normal developmental programs.

Indeed, these studies raise questions concerning the developmental origin of neurons innervating the heart. Although it is known that the NCCs contribute neurons to the arterial pole of the heart [47]–[49], the origin of these neurons and the mechanisms regulating their development are poorly understood. The NCCs migrating to the arterial pole of the heart were thought to possess limited neurogenic potential [50]. Nonetheless, a subset of NCCs migrating to the caudal pharyngeal arches is thought to contribute to the cholinergic cardiac ganglia within the parasympathetic plexus [51]. These neurons are initially observed at E11.5, but are not consistently and robustly detectable until E12.5 [50]. We observe noradrenergic ganglia, identified through their expression of TH and *DBH*, in the APCs of *Twist1* mutant embryos at E11.5. The presumptive NCC progenitors of these neurons, identified through *Sox10*, *Phox2b*, *Ascl1*, *Hand2* and *Hand1* expression, are evident in the E10.5 *Wnt1-Cre* CKO OFTs a day earlier, and in a much larger proportion of NCCs. It is notable that not all of the NCCs occupying the *Twist1* mutant APCs *trans*-differentiate into neurons. As stated above, the only established distinguishing characteristic of this subpopulation of cells is that they express the two *Hand* genes, while the remaining, ostensibly unaffected cells do not (Figure 2B; [14]). The significance of this observation is not clear, as Hand factors are not required for the differentiation of these ectopic neurons. Furthermore, it is unclear whether this *trans*-differentiation constitutes a corruption of endogenous neuronal differentiation pathways, or whether within a specific subpopulation of NCCs migrating to the APCs, which are competent to differentiate into neurons, Twist1 functions to repress neurogenic pathways, preventing them from differentiating in such a manner. It would thus be of interest to further explore the differences inherent in NCCs competent to differentiate into neurons in the APCs, and those that are not.

The Twist-box domain of Twist1 interacts with Runx2, Runx3 and Sox9 [18], [37], [46]. These interactions repress the respective functions of these non-bHLH factors by interfering with their DNA binding [18], [37], [46]. Here, we show that Twist1 (via the Twist-box domain) can both interact with the potent trans-activator of *Phox2b* transcription Sox10 and inhibit transcriptional activation of the *Phox2b* promoter (Figure 5A–5D). This repression is not mediated through simple interference with Sox10 DNA-binding to the *Phox2b* promoter (Figure 5G). Rather, Twist1 itself can bind to conserved, non-canonical E-box elements within the *Phox2b* promoter in a manner that is Twist-box dependent (Figure 5I). This finding is the first evidence that Twist-box mutations can influence Twist1 DNA binding. Co-immunoprecipitation experiments indicate that the Twist1 S192P mutation does not compromise Twist1 homodimerization, (J. Vincentz and A. Firulli, unpublished results). This suggests that this impaired DNA binding is not the result of defective Twist1 dimerization, but may instead result from a conformational change to the Twist1 secondary structure that impairs juxtaposition of the two required basic DNA-binding domains within the Twist1 S192P homodimer. It should also be noted that the *cis*-element to which Twist1 binds does not conform to a typical E-box sequence. The sequence, CAGGTT, is a putative binding site for the zinc-finger transcriptional repressor Snai1 (Snail) [52]–[54]. Snai1 and Twist1 can genetically interact in Drosophila and mouse [55], [56]. Like Twist1, Snai1 and the related factor Snai2 (Slug) is a crucial regulator of epithelial-mesenchymal transition (EMT) [12]. Indeed, Snai1/Snai2, in the presence of Sox9, is sufficient to induce an EMT in neural epithelial cells [57]. Thus, functional interactions between both Twist1 and Snai1/Snai2 with Sox proteins during NCC development would be an intriguing avenue of further study.

Phox2b is both necessary and sufficient to drive the SGN cell fate program [22], and *Phox2b* activation is dependent upon Sox10 [17]. The *Twist1^CC^* mouse model shows that disruption of the Twist-box is sufficient to promote cNCC *trans*-differentiation. Normal cNCC fate choice is partially restored when *Phox2b* gene dosage is reduced to heterozygousity and completely rescued when Phox2b function is completely ablated, demonstrating its necessity to initiate SGN cell fate. Additionally, Twist1 can interact with the bHLH factor Ascl1, as determined via co-immunoprecipitation analysis (data not shown). Collectively, Twist1 then interacts with at least five of the key SGN cell fate transcriptional regulators (Figure S9). Given that both cNCC and SGN NCC transcriptional programs are initiated by Bmp-signaling, and that these two programs, at least in part, share key transcriptional regulators (Hand1, Hand2, Gata3; [58], Sox4 [59], and (likely) Sox11 [60], [61]), it is obvious that a switch to ensure that the correct cell fate is specified and maintained is built into these developmental programs. Twist1 is a strong candidate to fulfill such a role.

Indeed, when Twist1 is expressed throughout the *Wnt1-Cre*-expressing NCCs, we observe a reduction in the differentiation of SGNs as indicated by the mutual exclusivity of Myc-Twist1 and TH immunoreactivity, decreased expression of the pan-neuronal marker Tubb3 and loss of expression of both *Hand2* and, more importantly, *Phox2b*. Additionally, the majority of the cells remaining in these hypoplastic ganglia express *Sox10* (Figure 8H, 8I). We can draw two conclusions from this observation. First, the presence of these *Sox10*-positive cells, along with that of Myc-Twist1-positive cells (Figure 8B, 8C), confirms that NCC-derived progenitors retain the ability to migrate properly to the region proximal to the dorsal aorta when Twist1 is ectopically expressed. Second, as *Sox10* is initially broadly expressed in NCC-derived neural precursors, but, by E12.5, is largely restricted to the surrounding support cells, we can infer either that ectopic Twist1 expression in NCCs specifically disrupts sympathetic neurogenesis, but not gliogenesis, or that *Sox10* is not downregulated in Twist1-overexpressing NCC-derived neural precursors, and these cells therefore fail to differentiate. In either case, this data clearly demonstrates that ectopic Twist1 expression in neuronal precursor cells fundamentally impairs sympathetic neurogenesis, and potentially maintains these cells in a precursor state.

It is intriguing to speculate about the insight these findings may provide into the role of TWIST1 in cancer. Reactivation of TWIST1-regulated embryonic programs has been proposed to contribute to tumor progression [62]. TWIST1 induces EMT in tumor cells, and thus plays a dominant role in defining the metastatic potential of primary tumors [63]. In both embryonic and adult stem cells, Twist1 is also thought to prevent differentiation, thus promoting a stem cell-like phenotype [13]. Thus, Twist1 represents a single factor that can intimately link two cellular processes, EMT and maintenance of a stem cell-like fate, both integral to tumor cell progression. As our data demonstrates that Twist1 can functionally antagonize key regulators of a sympathetic neuronal identity, it would be of interest to closely examine the function of TWIST1 in neuroblastomas and other tumors affecting neuronal derivatives of NCCs. TWIST1 overexpression in *N-Myc*-amplified neuroblastomas has been shown to inhibit p53-dependent apoptosis [64]. Pheochromocytomas are neoplasms originating from NCC-derived adrenal chromaffin cells. Chromaffin cells have a molecular profile similar to that of SGNs. Like SGNs, chromaffin cells do not normally express *Twist1*. TWIST1 is frequently upregulated in pheochromocytomas and, intriguingly, this aberrant expression is tightly associated with malignancy in these tumors [65]. Thus, the model presented here, by which Twist1 represses neuronal cell identity in NCC derivatives, may ultimately shed light upon the role of TWIST1 in cancer.

## Materials and Methods

### Transgenic mice

Genotyping of the *Twist1^tm1Bhr^* (*Twist1*; [66]), *Twist1^tm2Bhr^* (*Twist1^fx^*; [67] provided by James Martin), *Tg(Wnt1-cre)11Rth* (*Wnt1-Cre*; [68]), *Hand1^tm1.1(EGFP/cre)Abfi^* (*Hand1^eGFPCre^*; [16]), *Hand2^tm1Cse^*, (*Hand2^fx^*; [32]), *CAGCAT-Twist1*; [40], *Hand1^SG^-hsp68-lacZ*; [34], *Hand1^tm2Eno^* (*Hand1^lx^*; [69] provided by Eric Olson) and *Gt(ROSA)26Sor^tm1Sor^* (*R26R^lacZ^*; [70] alleles was performed as described. A 125bp region containing the mutated nucleotide in the *Twist1^Ska10^* (*Twist1^CC^*; [18]) allele was amplified using the primers Twist1CC(F), 5′-ACGAGCTGGACTCCAAGATG-3′, and Twist1CC(R), 5′-GGAGCTCCGCTGCTAGTG-3′. Amplicons were then purified and sequenced. *Phox2b^tm1Jbr^* (*Phox2b^lacZ^*; [71] provided by Michelle Southard-Smith and Jean-FranÇois Brunet) mice and embryos were genotyped using the primers Phox2bEx2(F), 5′-GTTCAGTGGCCCTTCACATC-3′, Phox2bEx2(R), 5′-TCCTCTCACGGGACACTTCT-3′, and lacZ_5′_out, 5′-CGGAAACCAGGCAAAGCGCC-3′ to generate ∼500 bp WT and ∼250 bp mutant amplicons.

### Histology

Alcian Blue, Nuclear Fast Red, Hematoxylin and Eosin (H&E) staining were performed as described [14], [72]. X-gal staining was performed as described [16].

### *In situ* hybridization

1Section *in situ* hybridizations were performed on 10 µm paraffin sections as described [14], [72]. Antisense digoxygenin-labeled riboprobes were synthesized using T7, T3 or SP6 polymerases (Promega) and DIG-Labeling Mix (Roche) using the following plasmid templates: *Twist1* (provided by Richard Behringer), *Hand1*, *Hand2*, *Sema3c*, *Runx1*, *Hey2* (provided by Yasuhide Furuta), *Sox9* (provided by Benoit De Crombrugghe), *Sox10* (provided by Paul Trainor), *Ascl1* (provided by Xin Zhang), *Phox2b*, *VaCHT* (provided by Peter Cserjesi), *Gata3*, *Ret*, *DBH* (provided by Jean-FranÇois Brunet), *PlexinA2*, *Pdgfrα*, *Smad6* (provided by Jonathan Epstein), *TrkA* (provided by David Ginty), *Brn3a*, *NeuroD1* (both provided by Eric Turner), and *ChAT* (IMAGE clone #8734071). Morphometric analyses of *Ascl1* staining were performed as described [73].

### Immunohistochemistry

Immunohistochemistry was performed as described [73] α-Tubb3 (AbCam), α-TH (AbCam), and α-Myc (Sigma) antibodies were used in combination with DyLight secondary antibodies (Thermo Scientific).

### Immunoblotting and co-immunoprecipitation experiments

Co-immunoprecipitation experiments were performed in HEK 293 cells using α-Myc and α-Flag (Sigma) as described [73]. Rat Sox10 was affixed with an N-terminal 6X Myc-tag via cloning into pCS2+MT. Densitometry analyses were performed using BioRad Quantity One software.

### Transactivation assays

Luciferase assays were performed in HeLa cells using the dual luciferase assay kit (Promega) as per manufacturer's instructions. 2 µg total DNA (0.25 µg of either pCS2-FLAG, pCS2-FLAG+Phox2b, or pRK5-FLAG+Sox10 (provided by Brian Black), 0.5 µg of either pcDNA3.1, pcDNA3.1-FLAG+Twist1[WT], or pcDNA3.1-FLAG+Twist1 S192P, 1.25 µg of PHOX2b(HindIII/NcoI)-pGL3b (provided by Diego Fornasari), and 0.125 µg of pRL-CMV) was transfected in 6-well plates using X-tremeGENE HP transfection reagent (Roche). Cell lysates were read using a 96-well micro-titer plate luminometer (Thermo Labsystems). Data represent four independent experiments. Error bars denote standard error.

### Bioinformatics

All sequences were obtained via Ensembl BLASTN search (http://www.ensembl.org) using the human *PHOX2b* 5′ promoter as a point of reference. PATTERNMATCH and CLUSTALW analyses were performed using the SDSC Biology WorkBench (http://workbench.sdsc.edu),

### Electrophoretic mobility shift assay (EMSA)

EMSAs were performed as previously described [74] with minor alterations. *In vitro* transcription and translation of Sox10 and Twist1 mRNAs were performed using pCS2-MT+Sox10 and pCS2-MT+Twist1 expression plasmids and the TnT rabbit reticulocyte lysate *in vitro* transcription system (Promega) as per manufacturer's instructions. 5 µL of TnT was used per reaction. Radiolabeled, annealed probes were purified using mini Quick Spin Oligo Columns (Roche). 1 µg poly(dG-dC) was used as a nonspecific DNA-binding competitor. Reactions were incubated for 30 min at room temperature following addition of probe. The following oligos, annealed to their complements, were used: E-Box 1, 5′-ACACTCTTACAAAA**CAGGTT**TTCTATGACATCAAGGTTTCTTC-3′; E-Box 1(Mut), 5-ACACTCTTACAAAA**tgGGac**TTCTATGACATCAAGGTTTCTTC-3′; HMG 3 5′-TTTCCAAGTAGTGT**GATTGAA**TTAAAGGGCAGGGA-3′; HMG 4 5′-TGGTATTAAATTCTAATTAGAGATGCAGGA**ATCAATG**ATAGGGAGGTTGGACAGCT-3′; E-box 2, 5′-AAGACCAACCGCTTTGCTATTGTC**CAAGTG**GAAAGAGCCAAGTTTATTATGAGG-3′. An oligo featuring a mutated HMG Box (HMG 4(Mut) 5′-TGGTATTAAATTCTAATTAGAGATGCAGGAA**TatcTGA**TAGGGAGGTTGGACAGCT-3′) was used as an unlabeled competitor.

### Ethics statement

Animal work (mouse) was performed according to an approved animal protocol from the University of Indiana IACUC, which is an AAALAC accredited program. We strive to focus on the three Rs (reduction/refinement/replacement) when working with animal models.

## Supporting Information

Figure S1 The *Wnt1-Cre* allele effectively ablates *Twist1* expression in cNCCs. *In situ* hybridization shows that *Twist1* expression is lost in the cNCC-derived pharyngeal arch and APC mesenchyme (compare arrowheads in A and B), but not the endocardium (compare black arrows in A and B) in E10.5 *Twist1^fx/−^*;*Wnt1-Cre(+)* embryos.
(TIF)

Figure S2 Abnormal NCCs in *Twist1*;*Wnt1-Cre* CKOs fail to express ecto-mesenchymal, markers. *In situ* hybridization shows that *PlexinA2* (A, B), *Smad6* (C, D), *Hey2* (E, F), and *Pdgfr*α (G, H) are excluded from the NCC aggregates (arrowheads) in E11.5 *Twist1^fx/−^*;*Wnt1-Cre(+)* embryos. Expression in surrounding ecto-mesenchyme is not noticeably affected (n = 3).
(TIF)

Figure S3 Ectopic expression of sympathetic neuron markers in mid- and late-gestation *Twist1^fx/−^*;*Wnt1-Cre(+)* mutant embryos. A–E) Section *in situ* hybridization reveals that ectopic *Phox2b* (A, B) and *Ascl1* (C, D) mRNA expression is detectable in the forming aorticopulmonary septum and aorticopulmonary cushions of *Twist1^fx/−^*;*Wnt1-Cre(+)* mutants concurrent with cNCC invasion at E10.5 (B, D). E–P) Marker analyses at E16.5 demonstrate that, in *Twist1^fx/−^*;*Wnt1-Cre(+)* mutants (G, H, K, L, O, and P), but not Control littermates (E, F, I, J, M, and N), robust ganglia (arrowheads), positive for *Phox2b* (G, H), *Hand2* (K, L), and TH (O, P) follow the aortic arch to the myocardial cuff.
(TIF)

Figure S4 Ectopic neurons in *Twist1*;*Wnt1-Cre* CKO APCs do not express markers that are not also expressed in sympathetic neurons. *In situ* hybridization shows that E11.5 *Twist1^fx/−^*;*Wnt1-Cre(+)* aggregates (arrowheads) do not express the sensory neuron markers *TrkA* (A, B), *Brn3a* (C, D), *NeuroD1* (E, F), and *Runx1* (G, H). The aggregates do express the cholinergic neuron markers *Ret* (I, J) and *VaCHT* (K, L), as do early sympathetic neurons; however, the parasympathetic neuron-specific marker *ChAT* (M, N) is not detectable above background levels in the aggregates. (n = 3)
(TIF)

Figure S5 OFT defects in *Twist1* mutants. A–E) Lineage trace analyses performed via X-gal staining (blue) of the *ROSA26R* β-galactosidase reporter allele show *Wnt1-Cre*-mediated recombination in the dorsal lip of the neural tube and all NCCs (A, B), whereas *Hand1^eGFPCre^*-mediated recombination is restricted to post-migratory NCCs in the AoPS and APCs (C, D). A schematized timeline (E) approximates when during development each allele initiates Cre recombination. F–M) Morphological and histological analyses at E16.5 show that *Twist1^fx/−^*;*Wnt1-Cre(+)* mutants display Persistent Truncus Arteriosus (PTA; G, asterisk) with an associated Ventricular Septal Defect (VSD; K, arrowhead). *Twist1*;*Hand1^Cre^* CKO aortic arches are largely indistinguishable from controls (compare F and H). Although PTA and Double Outlet Right Ventricle (DORV)+VSD (L, arrowhead) do rarely appear, the majority of hearts are grossly phenotypically normal. *Twist1^CC/CC^* homozygous mutant hearts are grossly phenotypically normal (compare F and J to I and M, respectively). BCT, brachio-cephalic trunk; LCC, left common carotid artery; LSA, left subclavian artery; LV, left ventricle; RCC, right common carotid artery; RSA, right subclavian artery; RV, right ventricle.
(TIF)

Figure S6 Twist1 regulates *Sema3c* in cNCCs in a Twist-box independent manner. The semaphorin family ligand *Sema3c* is expressed in post-migratory cNCCs (A, white arrowhead), and its downregulation is often associated with cNCC dysfunction. *Sema3c* mRNA is undetectable in the APCs of E11.5 *Twist1^fx/−^*;*Wnt1-Cre(+)* mutants, although myocardial *Sema3c* expression is unaffected (B, n = 9). *Sema3c* expression in post-migratory cNCCs is indistinguishable from controls (C, E) in all *Twist1^fx/−^*;*Hand1^eGFPCre^* mutants (D, n = 1/4), or in either *Twist1^CC/+^* heterozygotes (F, n = 4) or *Twist1^CC/+^* heterozygotes (G, n = 4).
(TIF)

Figure S7 Formation of ectopic SGN neurons in *Twist1* CKOs is Hand factor-independent. A–C) Hematoxylin and Eosin staining of E11.5 embryo sections shows that abnormal NCC condensations (arrowheads) are evident in the APCs of all *Twist1^fx/−^*;*Wnt1-Cre(+)* mutants, even when *Hand1* (A), *Hand2* (B), or *Hand1* and *Hand2* (C) function is lost.
(TIF)

Figure S8 The Twist-box domain of Twist1 is required for molecular interaction with both Phox2b, but does not inhibit Phox2b-mediated auto-regulation. A) Co-immunoprecipitation assays (n = 3) using epitope-tagged Twist1 and Phox2b show that Twist1 molecularly interacts with Phox2b, that a bHLH mutation of Twist1 (Twist1 T125;S127A) has no significant effect upon its ability to interact with Phox2b; however, mutation in the Twist-box domain (Twist1 S192P) impairs Twist1-Phox2b interaction (asterisk). B) Densitometry analyses quantitate these results. C) Transactivation assays of the human *Phox2b* promoter (n = 4) show that Phox2b auto-regulation is not significantly inhibited by Twist1 (p-value = 0.07) or the Twist1 S192P mutant (p-value = 0.16).
(TIF)

Figure S9 Summary of the role of Twist1 in NCC differentiation. In wild-type embryos, (A) NCC-derived SGN precursors respond to signaling from the dorsal aorta, upregulating a transcriptional cascade mediated by Sox10 and Phox2b and differentiating into TH- and DBH-expressing SGNs. (B) In the cNCCs, Twist1 functions to regulate OFT morphogenesis, presumably through a *Sema3c*-mediated mechanism, although this function likely reflects an early, pre-migratory Twist1 requirement. Additionally, Twist1 functions to repress ectopic neurogenesis in the OFT, by antagonizing the two factors that orchestrate NCC differentiation into neurons, Sox10 and Phox2b. (C) Loss of Twist1 function in the cNCCs prior to their migration leads to a loss to *Sema3c* expression, and associated OFT defects. Loss of *Twist1* or Twist-box function in post-migratory cNCCs leads to ectopic upregulation of *Sox10*, *Phox2b*, and *Ascl1* initiating a differentiation cascade which resembles that of SGNs, but which proceeds independently of Hand2 function, and is therefore distinct. (D) Ectopic Twist1 expression in all NCCs results in defective sympathetic neurogenesis, via its inhibition of Sox10 function and *Phox2b* transcription.
(TIF)

Table S1 OFT defects in *Twist1* mutants. The frequency of the OFT defects described in Figure. S5 is shown.
(DOCX)
