# Supplementary figures and images for: Twist1 Controls a Cell-Specification Switch Governing Cell Fate Decisions within the Cardiac Neural Crest
Source: PLoS Genet. 2013 Mar 21;9(3):e1003405. doi: 10.1371/journal.pgen.1003405 (PMC3605159; doi:10.1371/journal.pgen.1003405)

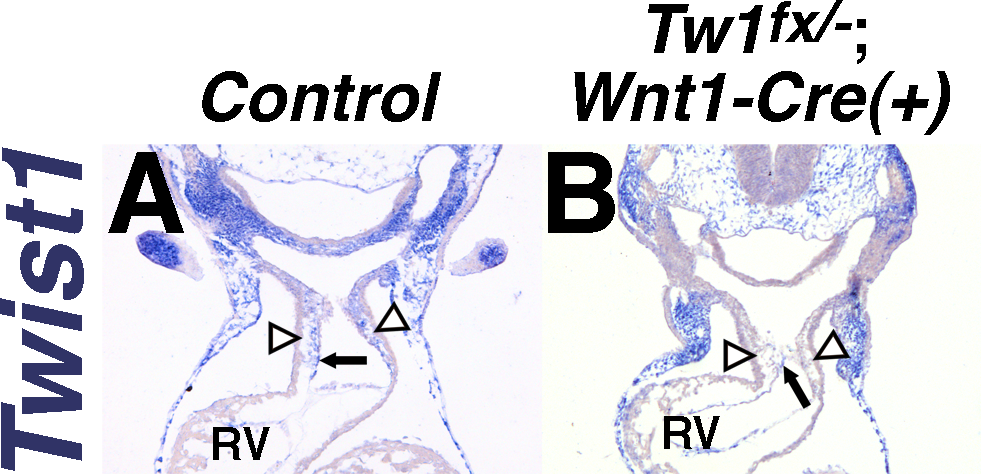

Supplement: Figure S1 — The Wnt1-Cre allele effectively ablates Twist1 expression in cNCCs. In situ hybridization shows that Twist1 expression is lost in the cNCC-derived pharyngeal arch and APC mesenchyme (compare arrowheads in A and B), but not the endocardium (compare black arrows in A and B) in E10.5 Twist1fx/−;Wnt1-Cre(+) embryos. (TIF) [file pgen.1003405.s001.tif]

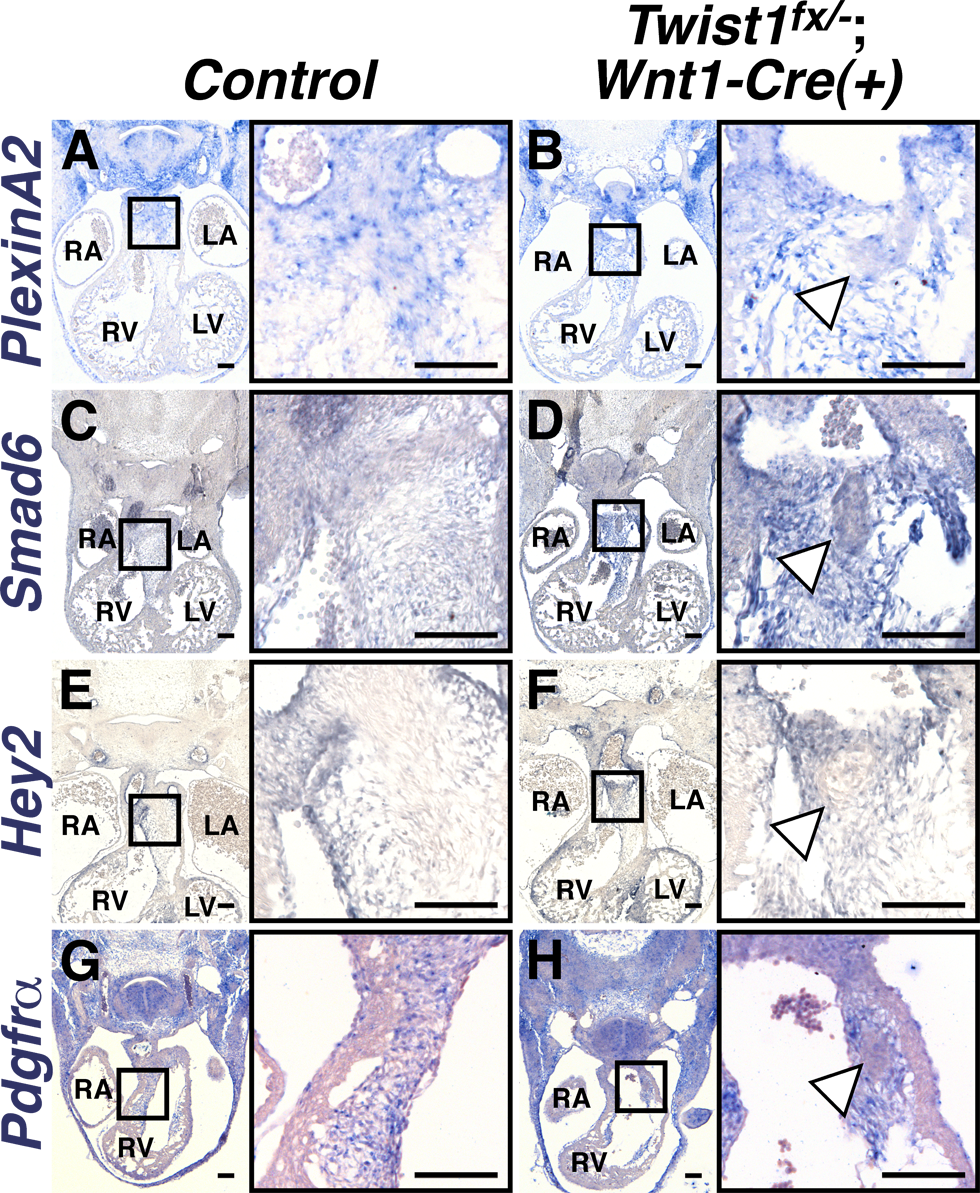

Supplement: Figure S2 — Abnormal NCCs in Twist1;Wnt1-Cre CKOs fail to express ecto-mesenchymal, markers. In situ hybridization shows that PlexinA2 (A, B), Smad6 (C, D), Hey2 (E, F), and Pdgfrα (G, H) are excluded from the NCC aggregates (arrowheads) in E11.5 Twist1fx/−;Wnt1-Cre(+) embryos. Expression in surrounding ecto-mesenchyme is not noticeably affected (n = 3). (TIF) [file pgen.1003405.s002.tif]

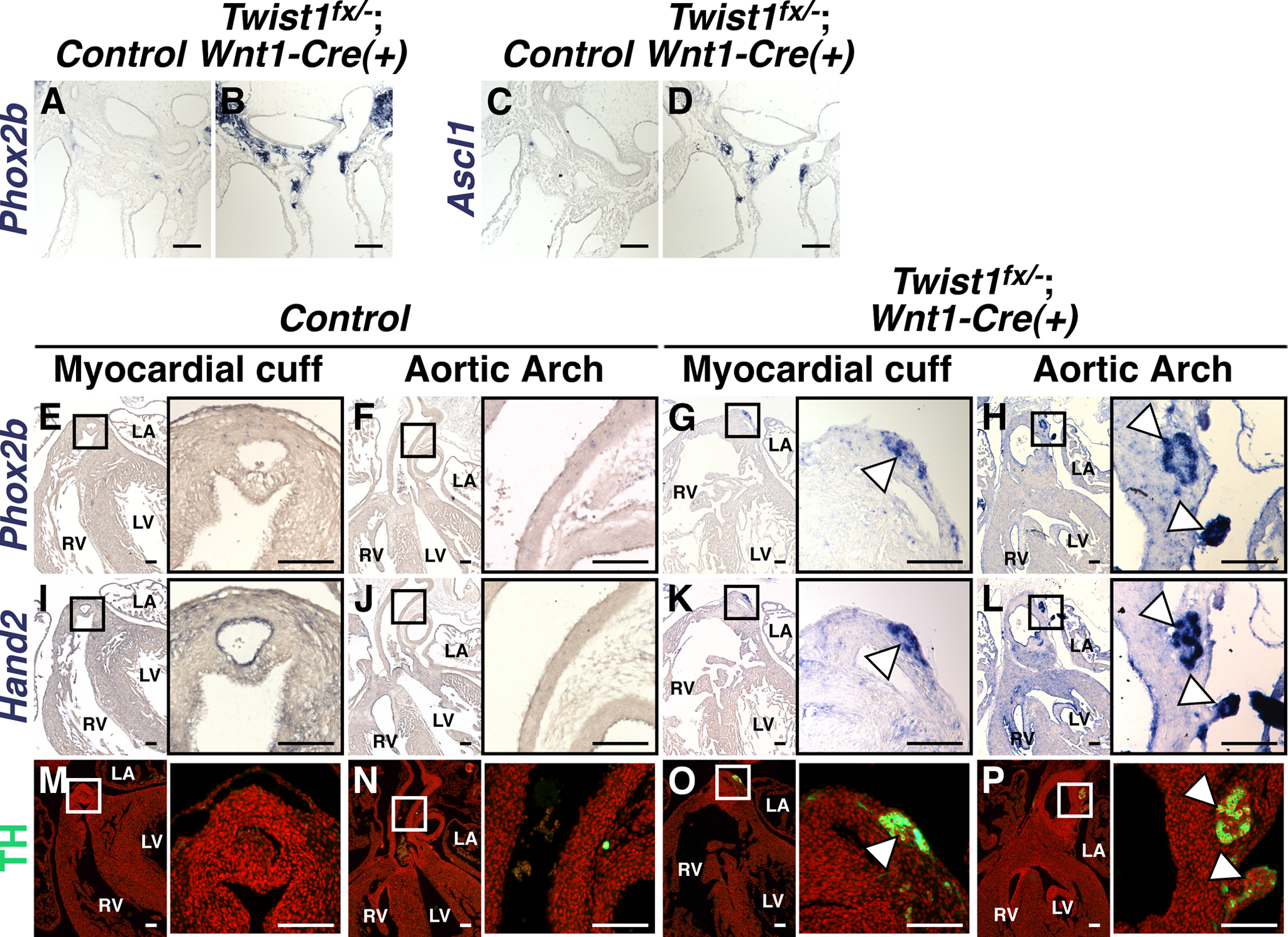

Supplement: Figure S3 — Ectopic expression of sympathetic neuron markers in mid- and late-gestation Twist1fx/−;Wnt1-Cre(+) mutant embryos. A–E) Section in situ hybridization reveals that ectopic Phox2b (A, B) and Ascl1 (C, D) mRNA expression is detectable in the forming aorticopulmonary septum and aorticopulmonary cushions of Twist1fx/−;Wnt1-Cre(+) mutants concurrent with cNCC invasion at E10.5 (B, D). E–P) Marker analyses at E16.5 demonstrate that, in Twist1fx/−;Wnt1-Cre(+) mutants (G, H, K, L, O, and P), but not Control littermates (E, F, I, J, M, and N), robust ganglia (arrowheads), positive for Phox2b (G, H), Hand2 (K, L), and TH (O, P) follow the aortic arch to the myocardial cuff. (TIF) [file pgen.1003405.s003.tif]

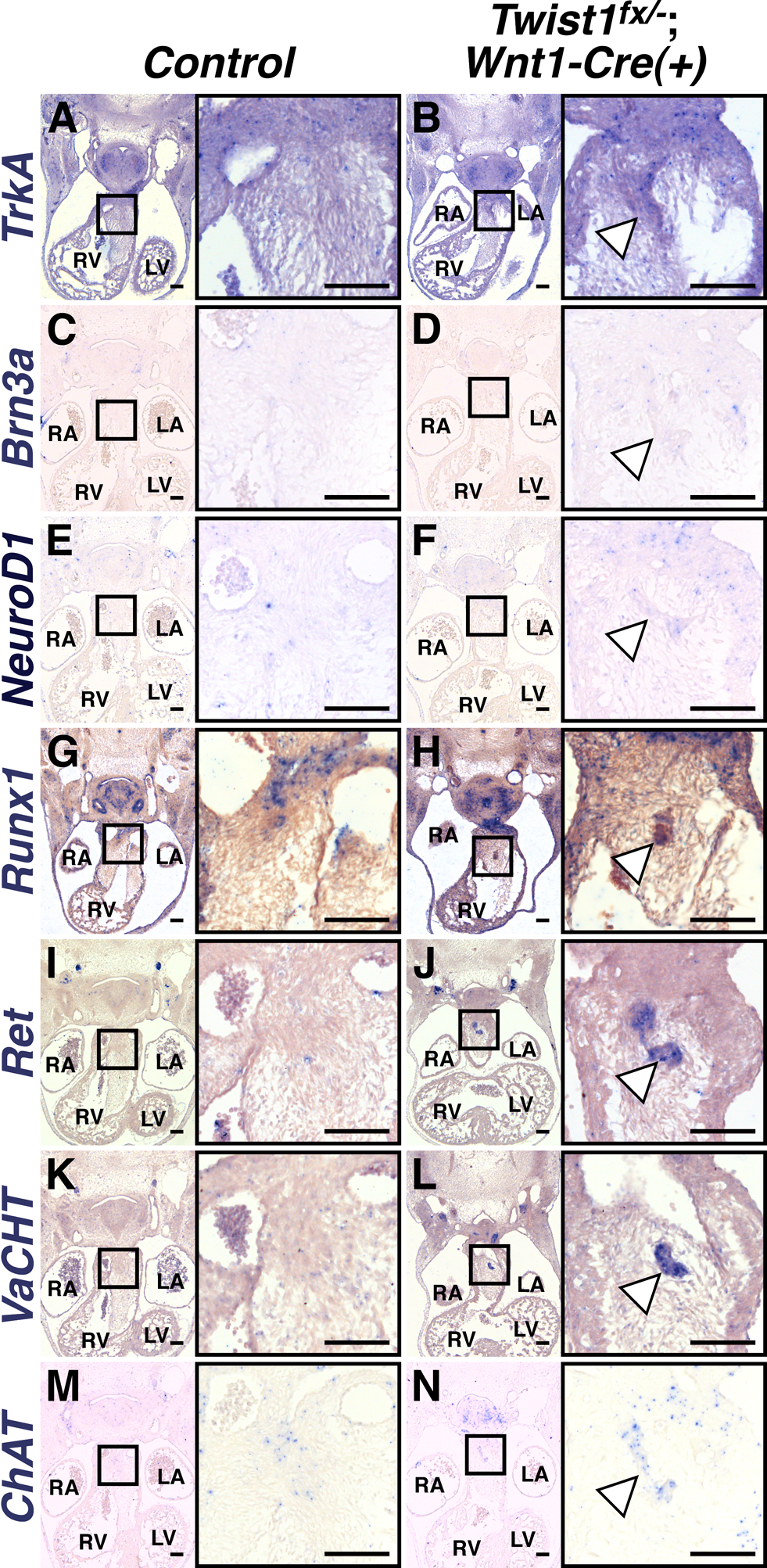

Supplement: Figure S4 — Ectopic neurons in Twist1;Wnt1-Cre CKO APCs do not express markers that are not also expressed in sympathetic neurons. In situ hybridization shows that E11.5 Twist1fx/−;Wnt1-Cre(+) aggregates (arrowheads) do not express the sensory neuron markers TrkA (A, B), Brn3a (C, D), NeuroD1 (E, F), and Runx1 (G, H). The aggregates do express the cholinergic neuron markers Ret (I, J) and VaCHT (K, L), as do early sympathetic neurons; however, the parasympathetic neuron-specific marker ChAT (M, N) is not detectable above background levels in the aggregates. (n = 3) (TIF) [file pgen.1003405.s004.tif]

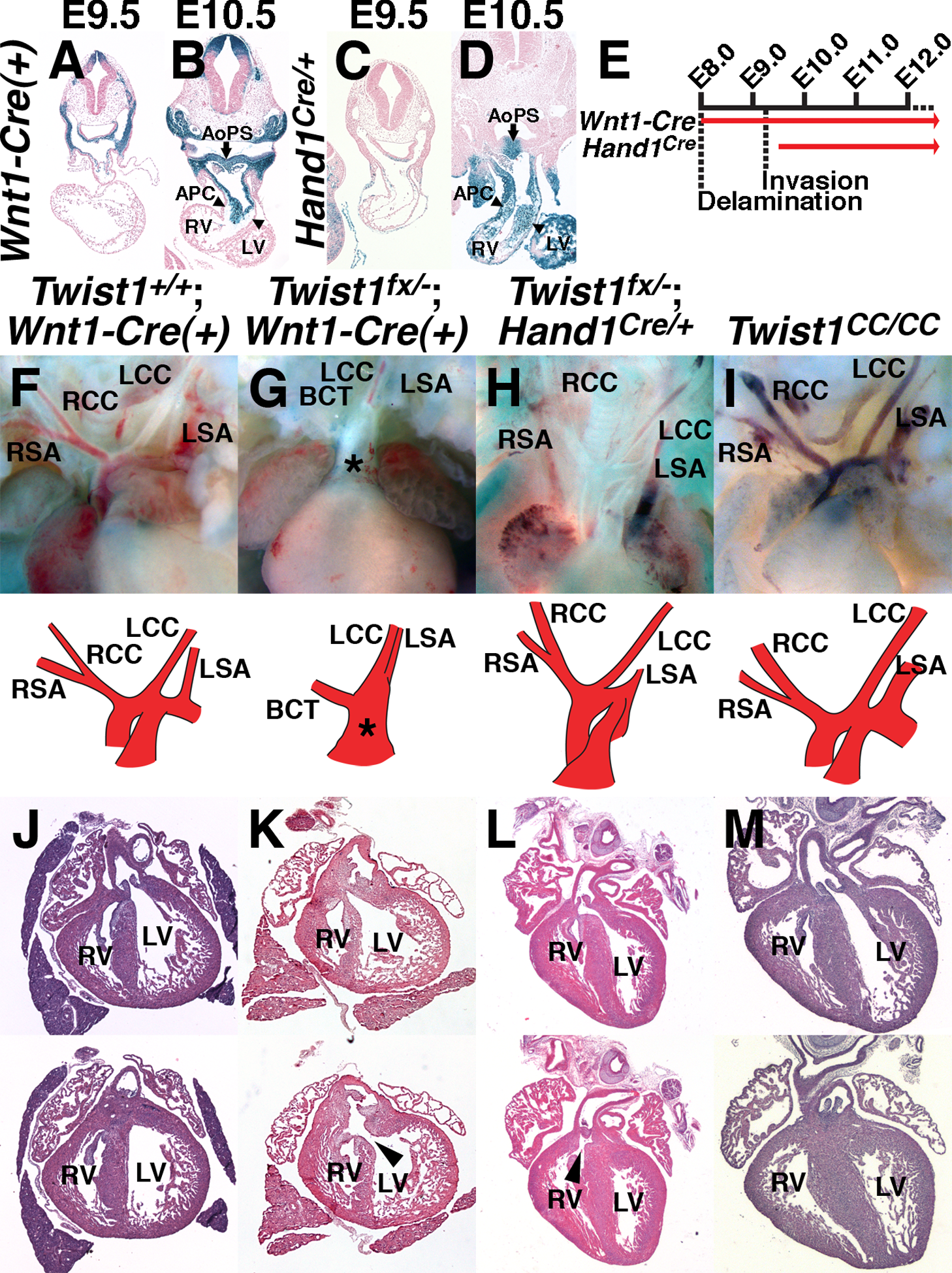

Supplement: Figure S5 — OFT defects in Twist1 mutants. A–E) Lineage trace analyses performed via X-gal staining (blue) of the ROSA26R β-galactosidase reporter allele show Wnt1-Cre-mediated recombination in the dorsal lip of the neural tube and all NCCs (A, B), whereas Hand1eGFPCre-mediated recombination is restricted to post-migratory NCCs in the AoPS and APCs (C, D). A schematized timeline (E) approximates when during development each allele initiates Cre recombination. F–M) Morphological and histological analyses at E16.5 show that Twist1fx/−;Wnt1-Cre(+) mutants display Persistent Truncus Arteriosus (PTA; G, asterisk) with an associated Ventricular Septal Defect (VSD; K, arrowhead). Twist1;Hand1Cre CKO aortic arches are largely indistinguishable from controls (compare F and H). Although PTA and Double Outlet Right Ventricle (DORV)+VSD (L, arrowhead) do rarely appear, the majority of hearts are grossly phenotypically normal. Twist1CC/CC homozygous mutant hearts are grossly phenotypically normal (compare F and J to I and M, respectively). BCT, brachio-cephalic trunk; LCC, left common carotid artery; LSA, left subclavian artery; LV, left ventricle; RCC, right common carotid artery; RSA, right subclavian artery; RV, right ventricle. (TIF) [file pgen.1003405.s005.tif]

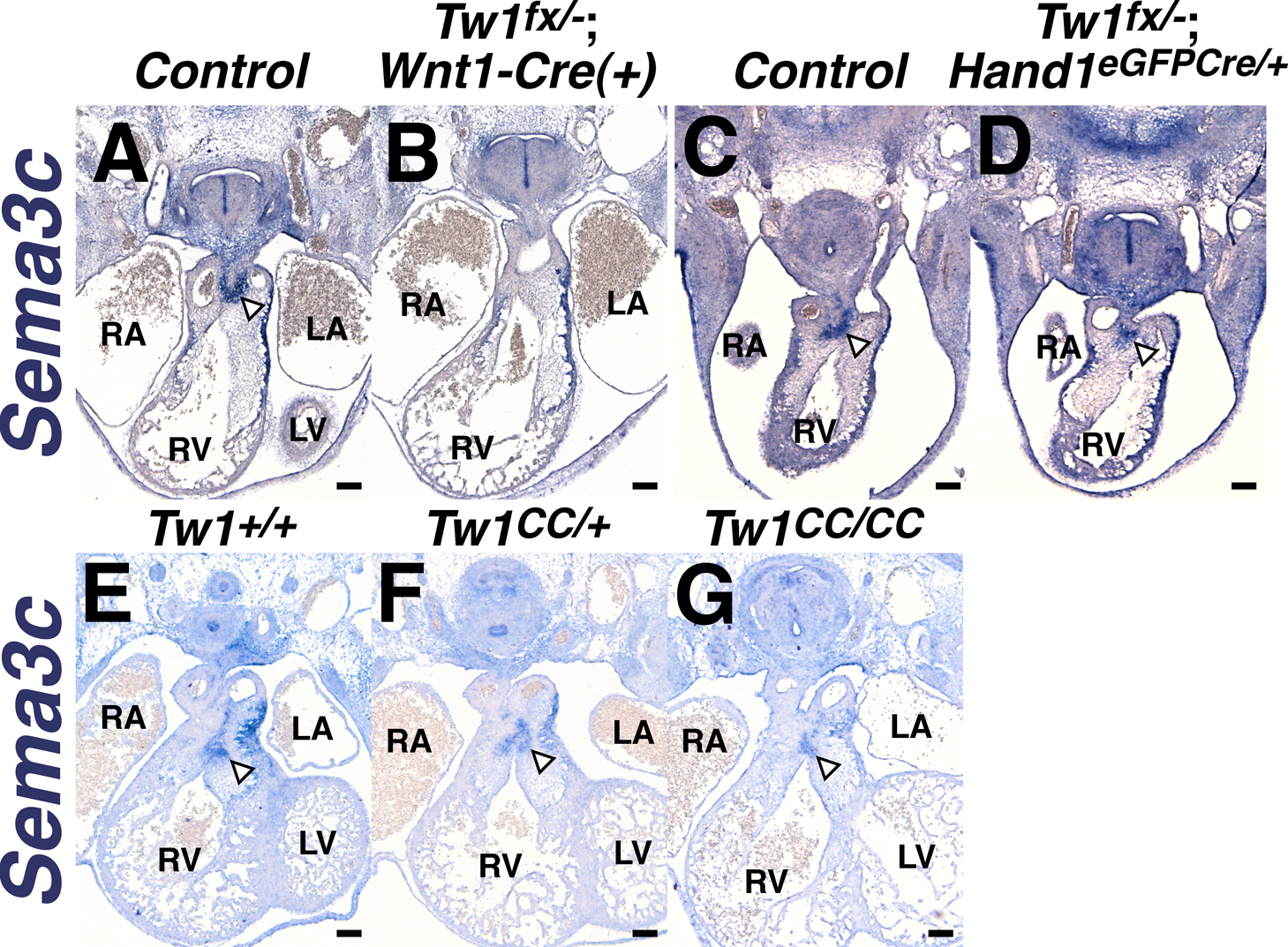

Supplement: Figure S6 — Twist1 regulates Sema3c in cNCCs in a Twist-box independent manner. The semaphorin family ligand Sema3c is expressed in post-migratory cNCCs (A, white arrowhead), and its downregulation is often associated with cNCC dysfunction. Sema3c mRNA is undetectable in the APCs of E11.5 Twist1fx/−;Wnt1-Cre(+) mutants, although myocardial Sema3c expression is unaffected (B, n = 9). Sema3c expression in post-migratory cNCCs is indistinguishable from controls (C, E) in all Twist1fx/−;Hand1eGFPCre mutants (D, n = 1/4), or in either Twist1CC/+ heterozygotes (F, n = 4) or Twist1CC/+ heterozygotes (G, n = 4). (TIF) [file pgen.1003405.s006.tif]

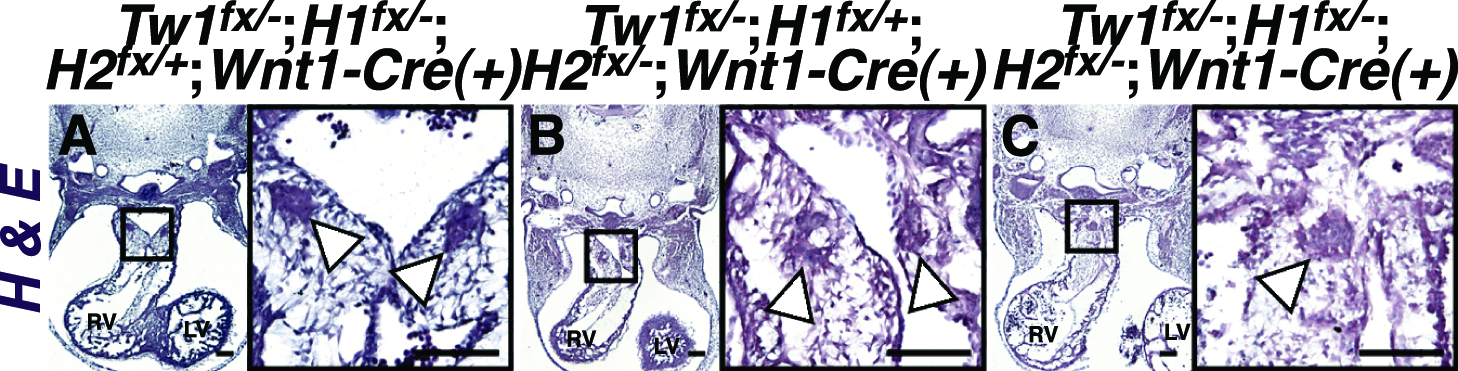

Supplement: Figure S7 — Formation of ectopic SGN neurons in Twist1 CKOs is Hand factor-independent. A–C) Hematoxylin and Eosin staining of E11.5 embryo sections shows that abnormal NCC condensations (arrowheads) are evident in the APCs of all Twist1fx/−;Wnt1-Cre(+) mutants, even when Hand1 (A), Hand2 (B), or Hand1 and Hand2 (C) function is lost. (TIF) [file pgen.1003405.s007.tif]

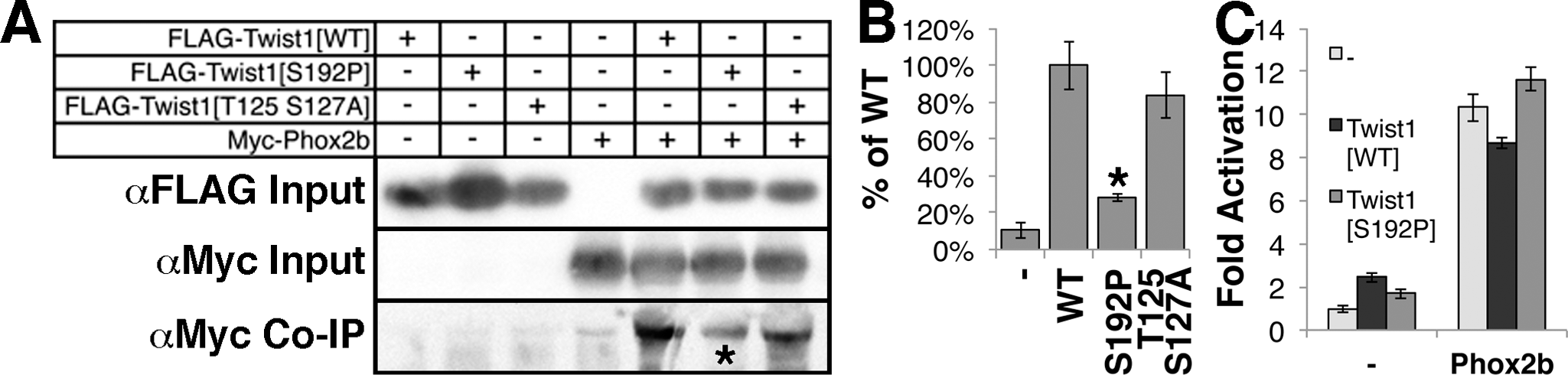

Supplement: Figure S8 — The Twist-box domain of Twist1 is required for molecular interaction with both Phox2b, but does not inhibit Phox2b-mediated auto-regulation. A) Co-immunoprecipitation assays (n = 3) using epitope-tagged Twist1 and Phox2b show that Twist1 molecularly interacts with Phox2b, that a bHLH mutation of Twist1 (Twist1 T125;S127A) has no significant effect upon its ability to interact with Phox2b; however, mutation in the Twist-box domain (Twist1 S192P) impairs Twist1-Phox2b interaction (asterisk). B) Densitometry analyses quantitate these results. C) Transactivation assays of the human Phox2b promoter (n = 4) show that Phox2b auto-regulation is not significantly inhibited by Twist1 (p-value = 0.07) or the Twist1 S192P mutant (p-value = 0.16). (TIF) [file pgen.1003405.s008.tif]

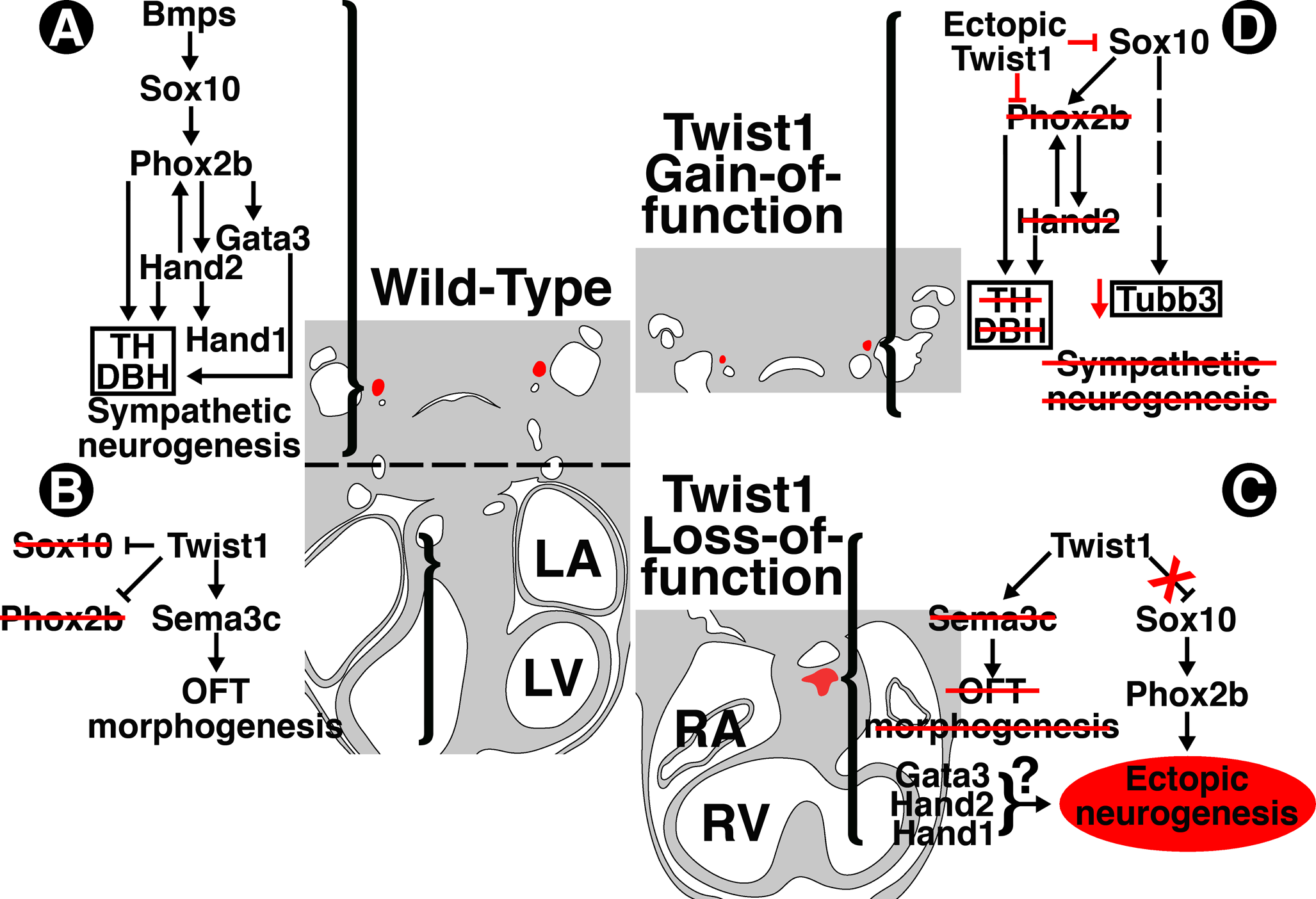

Supplement: Figure S9 — Summary of the role of Twist1 in NCC differentiation. In wild-type embryos, (A) NCC-derived SGN precursors respond to signaling from the dorsal aorta, upregulating a transcriptional cascade mediated by Sox10 and Phox2b and differentiating into TH- and DBH-expressing SGNs. (B) In the cNCCs, Twist1 functions to regulate OFT morphogenesis, presumably through a Sema3c-mediated mechanism, although this function likely reflects an early, pre-migratory Twist1 requirement. Additionally, Twist1 functions to repress ectopic neurogenesis in the OFT, by antagonizing the two factors that orchestrate NCC differentiation into neurons, Sox10 and Phox2b. (C) Loss of Twist1 function in the cNCCs prior to their migration leads to a loss to Sema3c expression, and associated OFT defects. Loss of Twist1 or Twist-box function in post-migratory cNCCs leads to ectopic upregulation of Sox10, Phox2b, and Ascl1 initiating a differentiation cascade which resembles that of SGNs, but which proceeds independently of Hand2 function, and is therefore distinct. (D) Ectopic Twist1 expression in all NCCs results in defective sympathetic neurogenesis, via its inhibition of Sox10 function and Phox2b transcription. (TIF) [file pgen.1003405.s009.tif]
