# Supplementary material for: Twist1 Controls a Cell-Specification Switch Governing Cell Fate Decisions within the Cardiac Neural Crest
Source: PLoS Genet. 2013 Mar 21;9(3):e1003405. doi: 10.1371/journal.pgen.1003405 (PMC3605159; doi:10.1371/journal.pgen.1003405)
Supplement: Table S1 — OFT defects in Twist1 mutants. The frequency of the OFT defects described in Figure. S5 is shown. (DOCX) [file pgen.1003405.s010.docx]

**Supplemental Table 1. OFT defects in *Twist1* mutants.**

| Genotype | n | PTA + VSD | Retroesophageal RSA | DORV + VSD | Phenotypically Normal |
| --- | --- | --- | --- | --- | --- |
| *Twist1^fx/-^*; *Wnt1-Cre(+)* | 5 | 5 (100%) | 1 (20%) | 0 (0%) | 0 (0%) |
| *Twist1^fx/-^*; *Hand1^eGFPCre/+^* | 7 | 1 (14.3%) | 0 (0%) | 1 (14.3%) | 5 (71.4%) |
| *Twist1^CC/CC^* | 8 | 0 (0%) | 0 (0%) | 0 (0%) | 8 (100%) |

DORV, double outlet right ventricle; PTA, persistent truncus arteriosus; RSA, right subclavian artery; VSD, ventricular septal defect.
